# Hydrophobic and Polarized Aromatic Residues Promote Internalization of Arg‐Rich Cell‐Penetrating Peptides through Ionpair‐π Interactions

**DOI:** 10.1002/chem.202501138

**Published:** 2025-07-04

**Authors:** Sonia Khemaissa, Antonio Bauzá, Émilie Lesur, Françoise Illien, Sandrine Sagan, Antonio Frontera, Astrid Walrant

**Affiliations:** ^1^ Sorbonne Université, École Normale supérieure PSL University CNRS, CPCV Chimie Physique et Chimie du Vivant Paris 75005 France; ^2^ Department de Química Universitat de les Illes Balears, Palma de Mallorca Baleares Spain; ^3^ Current Affiliation: ICMMO Université Paris‐Saclay Orsay France

**Keywords:** cell‐penetrating peptides, DFT, glycosaminoglycans, tryptophan

## Abstract

Cell penetrating peptides (CPPs) are small sequences that can cross cell membranes. Arg and Trp are highly prevalent amino acids in natural and synthetic efficient CPP sequences. In particular, Trp is essential and cannot be substituted by other hydrophobic or aromatic amino acids. The aim of the present study is to decipher the role of Trp in synthetic Arg/Trp CPP sequences. To do so, a small peptide library in which this residue was substituted by other natural or nonnatural amino acids was designed. Internalization of these peptides in cells was evaluated, and it appeared that combining aromaticity and hydrophobicity in the presence of Arg residues leads to enhanced internalization. The study of the interaction of these peptides with model lipid membranes revealed that the modulation of hydrophobicity promoted insertion in bilayers but had little impact on the binding affinity. On the other hand, more hydrophobic substitutes of Trp led to more favorable binding enthalpies to heparin. With density functional theory (DFT) analysis, we suggest that ion‐pair···π interactions between the aromatic ring and the ion pair formed by the positively charged Arg and the negatively charged cell surface groups can be established and could be at the origin of the unique internalization properties of Trp‐containing Arg‐rich CPPs.

## Graphical abstract

By introducing analogues of Trp in Ar/Trp cell‐penetrating peptides, we investigated the role of this unique amino acid in the cell uptake and interaction with cell membrane partners using experimental and computational approaches.

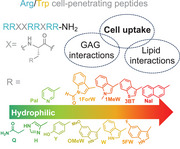

## 1 Introduction

Some biological processes, such as homeoprotein‐mediated paracrine cell communication, require that proteins enter cells to perform their biological activities without causing cell damage. Homeoproteins (HPs) are ubiquitous transcription factors from animals or plants, possessing a highly conserved homeodomain (HD) responsible for DNA interaction and membrane crossing. In the early 90s, the Prochiantz group identified the 3^rd^ helix of the HD of the Drosophila HP Antennapedia as being the minimal and sufficing sequence for cell internalization of the protein.^[^ ^1^ ^]^ This peptide, Penetratin, was the first identified amphipathic cell‐penetrating peptide (CPP), while the minimal Tat peptide, the other landmark CPP, isolated from the Tat protein of HIV in the same decade, is purely cationic.^[^ ^2^ ^]^ Penetratin and Tat are both relatively short sequences (16 residues for the former, 12 for the latter), and both carry a net positive charge, but otherwise present no striking common features, even though they share cell uptake and delivery properties. Many structure‐activity relationship studies were carried out to delineate structural requirements for efficient cell‐penetrating properties. The important role of Arg residues soon emerged and allowed the design of the first purely synthetic CPPs, oligoarginines (typically, Arg8, 9, or 10), which are a good mimic of Tat.^[^ ^3^, ^4^ ^]^ Regarding Penetratin, the Trp residue at position 48 in Antennapedia HD is conserved in more than 95% of 3^rd^ helices of HDs, and Trp56 is 34%.^[^ ^5^ ^]^ The substitution of both Trp48 and Trp56 by Phe in Penetratin results in an abolition of the internalization properties ^[^ ^1^ ^]^ whereas the single W48F substitution affects peptide translocation more drastically than the W56A substitution.^[^ ^6^ ^]^ Regarding cationic residues, a peptide variant where all Lys are replaced by Arg residues is more efficiently internalized.^[^ ^7^ ^]^ On those grounds, a simplified model hexadecapeptide, RW_16_, was proposed. RW_16_ recapitulates the amphipathic structure of Penetratin, was shown to enter cells, ^[^ ^8^ ^]^ and was used as a peptide delivery tool.^[^ ^9^ ^]^ A shorter, less charged, and less hydrophobic nonapeptide, R_6_W_3_ (RRWWRRWRR‐NH_2_), was subsequently designed.^[^ ^10^ ^]^ Comparison of uptake efficiency of Penetratin, Tat, R_9_, and R_6_W_3_ soon evidenced that R_6_W_3_ was a very promising model CPP sequence and that it was much better internalized than its ancestor Penetratin.^[^ ^11^ ^]^ Further studies on this sequence revealed that substitution of all three Trp by Leu (R_6_L_3_),^[^ ^12^ ^]^ Ala (R_6_A_3_)^[^ ^13^ ^]^ and Phe (R_6_F_3_)^[^ ^14^ ^]^ abolished the uptake properties of the peptide. The groups of Cory and Dhar also extensively studied R_6_X_3_ sequences (where X = W, R, A, L, and H) for the delivery of pDNA/peptide nanoparticles and showed that only R_6_W_3_ and R_9_ were efficient as transfection vectors,^[^ ^15^, ^16^ ^]^ which they correlated with a superior ability to insert into membranes.^[^ ^17^ ^]^ Trp was also replaced by nonnatural residues composed of a galactosyl unit coupled to the peptide by a triazole link,^[^ ^13^, ^18^ ^]^ which again was deleterious for cell‐delivery properties. On the other hand, a study from 2023 suggested that substitution of Trp by nonnatural constrained analogues in R_6_W_3_ could confer promising membrane interaction and uptake properties to the CPP.^[^ ^19^ ^]^ Finally, work from our group revealed that Arg addition in Arg/Trp nonapeptides could compensate for Trp loss and vice‐versa and that the least efficient combination was two Trp and seven Arg, regardless of their position in the sequence.^[^ ^20^ ^]^ Research from the Norden and Parang groups also showed that increasing the number of Trp residues in Arg‐rich CPPs had a great impact on their uptake.^[^ ^21^, ^22^ ^]^

Trp is a unique amino acid that is often encountered in membrane proteins^[^ ^23^ ^]^ and membrane‐active peptides.^[^ ^24^ ^]^ The bulky and aromatic indole side chain is hydrophobic but is also polar and has H‐bond donating properties and thus can engage in many types of biomolecular interactions.^[^ ^23^ ^]^ In particular, work from our group and several others showed that the presence of Trp in Arg‐rich CPPs promoted interactions with lipid membranes^[^ ^12^, ^25^ ^]^ and glycosaminoglycans (GAGs).^[^ ^26^ ^]^ This is particularly interesting, as the nature of extracellular binding partners of CPPs plays a major role in the subsequent internalization mechanisms.^[^ ^27^ ^]^ In the present study, we designed a small library of R_6_X_3_ peptides, where X are natural or nonnatural amino acids that recapitulate one or several properties of Trp: aromaticity, polarity, H‐bond donation, amongst which are several nonnatural Trp analogues carrying various modified indole rings. We evaluated their cytotoxicity and uptake efficiency in wild‐type and GAG‐deficient CHO cell lines and investigated their interactions with lipid membranes and GAGs in vitro. Our recent work showed that Trp could be involved in tripartite ion‐pair/π‐type interactions with the guanidinium moiety of Arg and carboxylates found on GAGs.^[^ ^20^ ^]^ Herein, DFT analyses were used to study how the structure of the R side chain would modulate such interactions.

## 2 Results

### 2.1 Peptide Design and Characterization

**Figure 1:**
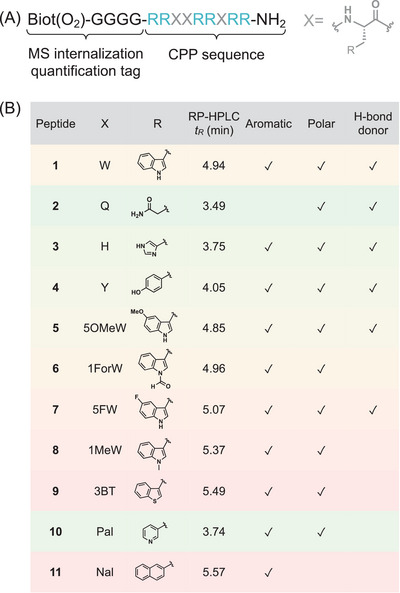
Peptide library. A) Peptide sequence, X being different natural or nonnatural L‐amino acids. B) Peptide list with the structure of the side chain on X, color‐coded from the most hydrophilic (green) to the most hydrophobic (red) sequence, and chemical properties.

Our peptide library was based on R_6_W_3_. All peptides were synthesized using the Boc/Bzl solid‐phase peptide synthesis strategy. They are amidated at their *C*‐terminus and carry an MS‐quantification tag consisting of four Gly and a biotin sulfone (Biot(O_2_)) at the *N*‐terminal position^[^ ^28^ ^]^ (Figure 1a). Our reference peptide **1** thus corresponds to the following sequence: Biot(O_2_)‐G_4_‐RRWWRRWRR‐NH_2_. The three Trp residues were then substituted by other natural amino acids: glutamine (Q) in peptide **2**, found in Tat and Penetratin, and aromatic histidine (H, peptide **3**) and tyrosine (Y, peptide **4**); by nonnatural l‐Trp analogues: 5‐Methoxy‐Trp (5OMeW, peptide **5**), 1‐Formyl‐Trp (1ForW, peptide **6**), 5‐Fluoro‐Trp (5FW, peptide **7**), 1‐Me‐Trp (1MeW, peptide **8**), 3‐Benzothienyl‐Ala (3BT, peptide **9**); and by other nonnatural l‐aromatic amino acids: 3‐Pyridyl‐Ala (Pal, peptide **10**) and 2‐Naphtyl‐Ala (Nal, peptide **11**). Their hydrophobicity was quantified according to their retention time (*t*_R_) in reverse‐phase analytical HPLC on a C18 analytical column. The *t*_R_ were consistent with the hydrophobicity of the different X amino acids as quantified by their logD at pI. Their main properties are summarized in Figure 1b. Full sequences and MALDI‐TOF MS experimental m/z are reported in Table S1.

### 2.2 Internalization Quantification

The quantification of internalization of these peptides was performed at 37 °C, a temperature at which CPPs are internalized by endocytosis and direct translocation concomitantly, using 10 µM extracellular peptide concentration and 1 hour incubation. Internalization was quantified in WT CHO cells (CHO‐K1) and GAG‐deficient CHO cells (CHO‐pgsA 745) to account for the role of GAGs in their uptake. Quantification was performed using a MALDI‐TOF‐based method, which relies on the use of an isotopically labeled internal standard and allows absolute quantification of the amount of peptide inside cells^[^ ^28^ ^]^ routinely used in our group. As a rule of thumb, 1 pmol intracellular peptide corresponds to 1 µM intracellular peptide concentration, provided an average cellular volume of 1 pL for CHO cells and quantification in 10^6^ cells.

**Figure 2:**
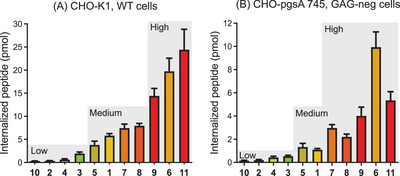
Quantification of peptide internalization for an extracellular concentration of 10 µM and 1 million A) CHO‐K1 cells and B) CHO‐pgsA 745. Values were averaged from three independent experiments performed in triplicate. Error bars represent the standard error of the mean. Peptides were classified as poorly internalized (“Low” grey shading) when the amount internalized was less than half of that of **1**, highly internalized (“High” grey shading) when more than twice the amount of **1**, and moderately internalized (“Medium” grey shading) in between.

In WT CHO‐K1, peptides can be divided into three groups with different internalization properties compared to **1** (Figure 2a). Poorly internalized peptides (less than half of **1**) correspond to **10**, **2, 3,** and **4**. Highly internalized peptides (two to three times more than **1**) encompass **9**, **6,** and **11**. Notably, for these peptides, the final intracellular peptide concentration was higher than the extracellular concentration. Finally, peptides with substituted indole side chains **5**, **7,** and **8** behave similarly to the reference [1].

In GAG‐deficient pgsA‐745 (Figure 2b), the overall amount of internalized peptide is reduced two‐to fivefold, but the general trend is roughly similar, with **9**, **11,** and **6** being the three best‐internalized peptides, together with **8** and **7** to a lesser extent.

These results show that GAGs are important for the internalization of these peptides, even though **6**, and **7** to a lesser extent, appear less impacted than other peptides. More importantly, they help delineate a few rules regarding successful Trp substitutions: 1/ H‐bond donation is not an important feature; 2/ substituents on the indole ring have little impact (except for 1ForW), 3/ aromaticity is not sufficient to promote internalization, and 4/ hydrophobicity is important. These points will be further discussed and put in perspective in the discussion section.

The internalization process induces a membrane perturbation that can evoke cytotoxicity should this perturbation be more than transient, as our library is composed of amphipathic peptides, some of which with quite hydrophobic residues. A cytotoxicity assay was performed in CHO‐K1 cells for different peptide concentrations (10 and 50 µM) for 1 hour of incubation. Most peptides were not or were little cytotoxic at the lower peptide concentrations (Figure S1). **9** appears to be cytotoxic in a dose‐manner dependence with 60% cytotoxicity for 50 µM extracellular concentration. A similar cytotoxic effect of the substitution of the three Trp in the antimicrobial peptide Tritrpticin by 3BT has previously been reported.^[^ ^29^ ^]^ This might be an off‐target effect, as benzothiophene is a dehydroreductase inhibitor,^[^ ^30^ ^]^ our viability assay being based on dehydroreductase activity in viable cells.

### 2.3 Interactions with Model Lipid Membranes

CPPs are part of the large family of membrane‐active peptides, and **1** being amphipathic and Trp‐rich, it should interact favorably with lipid bilayers. Indeed, our previous work showed that it bound to liposomes composed of negatively charged lipids such as phosphatidylglycerol (PG).^[^ ^12^ ^]^ **1** was also previously shown to insert into a DMPC/DMPG (80:20) monolayer, leading to a very slight increase in surface pressure (0.6 mN/m).^[^ ^13^, ^31^ ^]^ Herein, we quantitatively analyzed the binding of our R_6_X_3_ peptides with large unilamellar vesicles (LUVs) composed of anionic 1‐palmitoyl‐2‐oleyl‐PG (POPG) by isothermal titration calorimetry (ITC) and evaluated the insertion and effect of the peptides on multilamellar vesicles (MLVs) composed of dimyristoyl‐PG (DMPG) by differential scanning calorimetry (DSC). Finally, we evaluated the ability of poorly internalized peptide **2** and highly internalized peptides **6**, **9,** and **11** to insert into DMPC/DMPG (80:20) monolayers.

**Figure 3:**
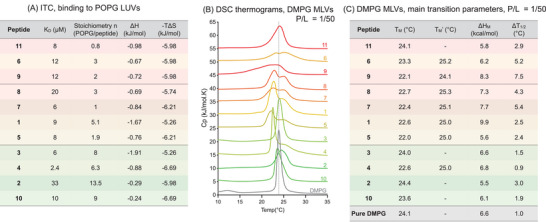
Interactions of R_6_X_3_ with anionic lipids studied by ITC A) and DSC B,C). A) Thermodynamic parameters obtained by ITC are averaged over two independent experiments. B) DSC thermograms are given for a peptide‐to‐lipid ratio (P/L) of 1/50; the thermogram obtained for pure DMPG is shown in grey; the thermograms were offset for easier comparisons. C) Thermodynamic parameters associated with the main transition on a heating scan. T_M_’ indicates the second maximum of the transition peak when splitting was observed.

The binding parameters derived by ITC (Figure 3a) reveal that all peptides bind POPG LUVs with very similar affinities of approximately 10 µM, which is consistent with previously reported values for R_6_X_3_ peptides.^[^ ^12^ ^]^ The process is largely entropy‐driven, as expected for peptides binding charged surfaces and thus releasing immobilized ions and water molecules. There seems to be no obvious correlation between the hydrophobic nature of the X amino acid and affinity. Although the stoichiometry values should be taken with caution, it is interesting to notice that **3** and **10** bind more lipids, which could be related to the fact that at pH 7.4, Pal and His are mostly under their cationic form.

While ITC gives interesting quantitative insight on the binding, DSC can provide complementary information on the effect of peptide binding and insertion within the membrane. Here, increasing amounts of peptide were added to DMPG MLVs, and the phase transitions of DMPG were monitored. Heating thermograms corresponding to peptide‐to‐lipid ratio of 1/50 are reported in Figure 3b, and the associated main transition parameters are in Figure 3c. The thermogram corresponding to the lipid alone shows two peaks (gray bottom curve). The first one, the pre‐transition peak, at 13 °C, corresponds to the transition from the gel phase L_β’_ to the rippled phase P_β’_, and the other one, at 24 °C, corresponds to the rippled‐to‐fluid‐phase L_α_ transition. In all cases, the addition of an R_6_X_3_ peptide leads to the disappearance of the pre‐transition peak, which is usually interpreted as an interaction occurring between the peptides and the lipid headgroups.^[^ ^32^ ^]^ On the other hand, the effect on the main transition peak highly depends on the peptide. **3** and **10** have the least obvious effect on the main transition peak. At pH 7.4, Pal and His are probably at least partially in their cationic form, and their effect on the transition is consistent with that of R_9_ reported in the literature.^[^ ^12^ ^]^ Most peptides lead to a decrease in the main transition temperature, suggesting a destabilization of the gel phase, interpreted as a “fluidizing” effect. Finally, most peptides induce a clear split in the main transition peak, in particular those with an indole‐like side chain. This has been attributed to an inhomogeneous distribution of the peptide on the bilayer and was previously reported for R_6_W_3_ and Penetratin. It is interesting to notice that analogues with no nitrogen atom in their aromatic ring (**9** and **11**) do not lead to this splitting. No obvious relationship between hydrophobicity and effect on the main transition temperature can be observed, but the most hydrophobic peptides always lead to peak broadening (ΔT_1/2_ in Figure 3c), which is consistent with the insertion of the peptides in the hydrophobic region of the bilayer. Notably, **6** has the strongest effect on the main transition peak and is also the one with the most efficient uptake in GAG‐deficient CHO pgsA‐745, in which lipids probably play a major role as binding partners.

Insertion of peptides **2, 6, 9,** and **11** in DMPC/DMPG (80:20) monolayers at the air/PBS interface in a Langmuir trough using the Wilhelmy method was evaluated by following the surface pressure. When injected in the PBS subphase, **6, 9,** and **11** induced a small surface pressure increase of 1 to 2 mN/m, slightly larger than what was reported for **1**,^[^ ^13^, ^31^ ^]^ whereas **2** led to no change in surface pressure, again consistent with previous observations,^[^ ^13^ ^]^ confirming the DSC observations (Figure S3).

### 2.4 Interactions with GAGs

**Figure 4:**
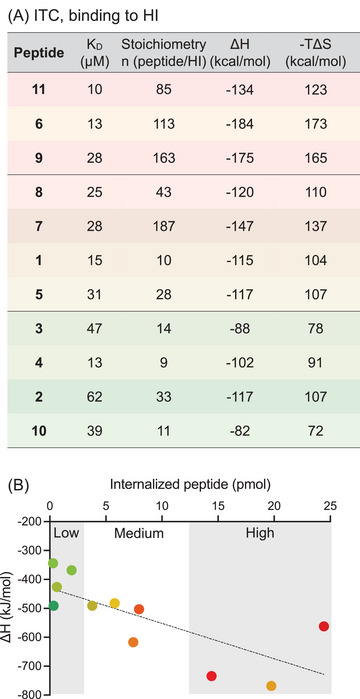
Interactions of peptides with HI studied by ITC. A) Thermodynamic parameters were obtained by titrating HI into the different peptides. Values are averaged over two experiments. B) Relationship between amounts internalized and binding enthalpies.

GAGs are prominent binding partner and, as shown herein, are important for the uptake of R_6_X_3_ peptides. As previously with lipids, we characterized the interaction of our peptides with GAGs using ITC. Heparin (HI), a highly sulfated GAG of 12 kDa, was used as a GAG mimic. The thermodynamic parameters of the interaction with GAGs are given in Figure 4a. As previously reported,^[^ ^26^ ^]^ the interaction is driven by enthalpy with very large negative ΔH values and is also very disfavored by entropy. This is not surprising, as the interaction we are studying involves mainly electrostatics and H‐bonding and probably greatly reduces the flexibility of the long polysaccharide. The measured K_D_ are all in the same range (10‐60 nM), and a very clear enthalpy/entropy compensation phenomenon was observed (Figure S2). Looking at the thermodynamic data in more detail, there are interesting observations to be made. In particular, for the highly internalized peptides **6, 9,** and **11**, the binding stoichiometries with heparin are up to 16 times higher than for **1**, making GAGs appear as peptide collectors at the cell surface. Still, this has to be taken with caution, as **7** has the highest peptide/HI ratio and is not the most successfully internalized. On the other hand, a negative cross‐correlation between stoichiometry and uptake for CPPs of the lactoferrin family,^[^ ^33^ ^]^ related to a better ability to cluster GAGs and trigger endocytosis.

The enthalpy of interaction is also a very striking feature. If only electrostatics/H‐bonding were involved, the measured ΔH should be similar for all peptides and probably larger for **3** and **10,** which carry more charges at pH 7.4. It is, however, not so, and strikingly, the most hydrophobic and better internalized peptides give the largest ΔH, up to 1.6‐fold more favorable than **1** (Figure 4b). This had already been observed with Arg/Trp CPPs in our previous study, with a more favorable binding enthalpy when increasing the number of Trp in the sequences.^[^ ^20^ ^]^ DFT analyses revealed that Trp could contribute favorably to the binding by establishing ion‐pair···π interactions with Arg residues and carboxylates on GAGs.

### 2.5 DFT Analysis

**Figure 5:**
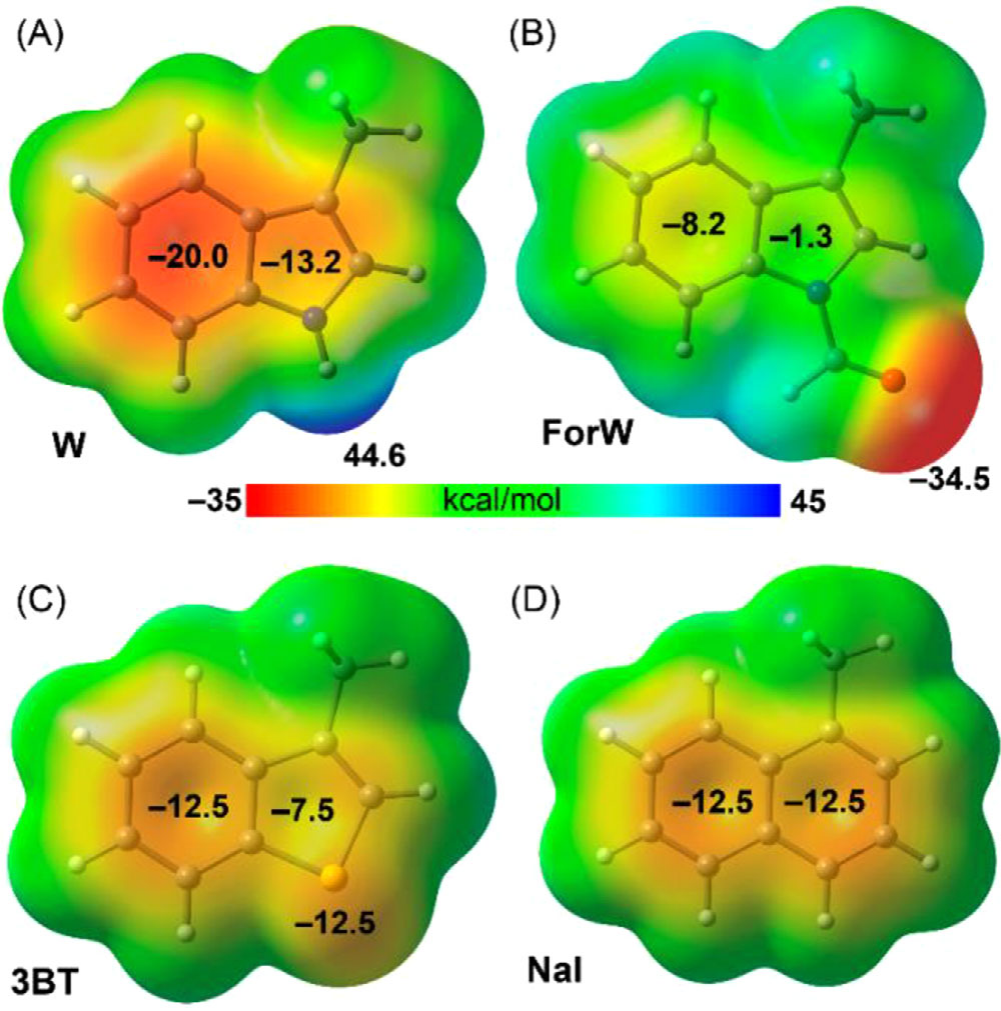
MEP surfaces of the side chain of W A), 1ForW B), 3BT C), and Nal D). Energies at selected points in kcal/mol.

First, we analyzed the molecular electrostatic potential (MEP) surfaces of the Trp side chain and its analogues with enhanced penetration ability: 1ForW, 3BT, and Nal. This analysis aimed to investigate their differences from an electrostatic perspective. The MEP plots, presented in Figure 5, reveal that Trp is a strong hydrogen bond donor, with a maximum MEP value of 44.6 kcal/mol at the NH group, and also exhibits a large negative MEP over the center of its six‐membered aromatic ring.

For the other molecules, the MEP values over the aromatic surface are significantly smaller, consistent with the higher hydrophobicity observed experimentally for 3BT and Nal. In the case of 1ForW, the reduction in MEP over the aromatic ring is even more pronounced. However, the MEP at the oxygen atom of the carbonyl group is significantly negative (−34.5 kcal/mol), indicating strong hydrogen bond acceptor ability (amidic nature) and explaining its lower hydrophobicity compared to 3BT and Nal. Notably, the experimental hydrophobicity of W and 1ForW is nearly identical (a difference of only 0.02 s in *t*_R_; see Figure 1), which can be attributed to the presence of either a strong hydrogen bond donor (W) or an acceptor (1ForW) group.

**Figure 6:**
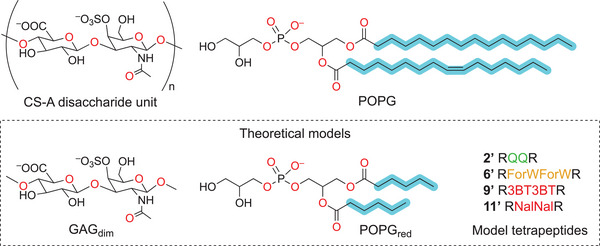
Theoretical modes of GAG, POPG, and CPPs used for simulations.

The interaction modes of CPPs with theoretical models of GAGs and POPG have been computed, along with their corresponding interaction energies. Figure 6 (bottom insert) presents the minimalistic models of GAG, POPG, and CPPs used in the DFT study.

For GAGs, we employed a monosulfated disaccharide, denoted as GAG_dim_, as a representative model of chondroitin 4‐sulfate (CS‐A), in which the bridging oxygen atoms were methylated. For POPG, we simplified the structure by truncating the long lipid chain. Finally, different tetrapeptides **2′**, **6′**, **9′,** and **11′** were used as representative models for CPPs.

**Figure 7:**
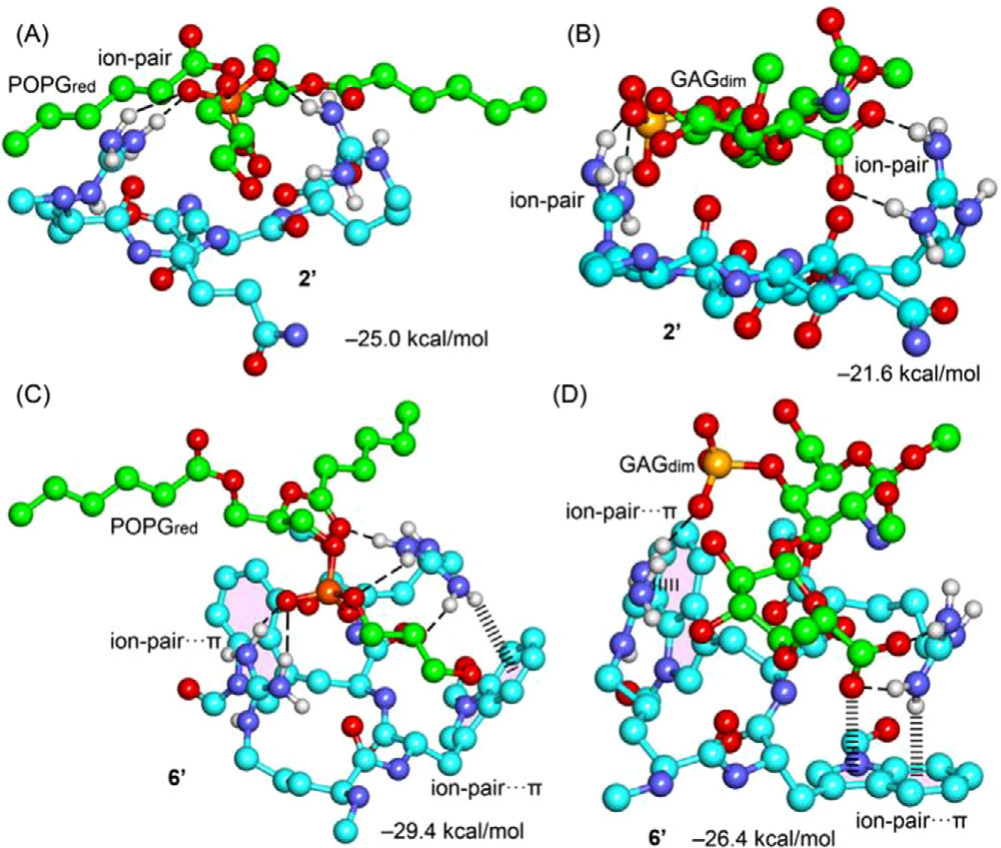
Optimized geometries of **2′**···POPG_red_ A), **2′**···GAG_dim_ B), **6′**···POPG_red_ C), and **6′·**··GAG_dim_ D) with indication of the ion‐pair (top) and ion‐pair···π interactions (bottom). The C‐atoms of the peptide motifs are colored blue, and those of POPG_red_ and GAG_dim_ are colored green. The ion‐pair distances are i) 3.406Å (O···C) in **2′·**··POPG_red_ complex and ii) 3.785Å (O···C) and 3.727Å (C···C) in the **2′**···GAG_dim_ complex.

**Figure 8:**
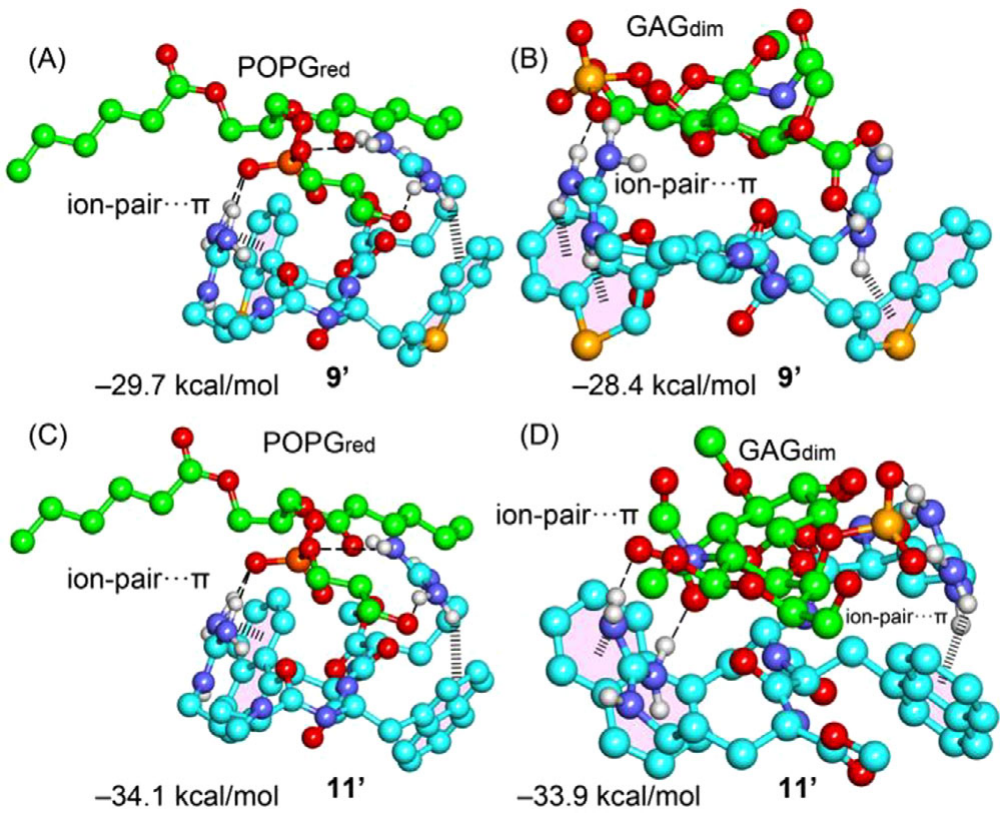
Optimized geometries of **9′**···POPG_red_ A), **9′**···GAG_dim_ B), **11′**···POPG_red_ C), and **11′**···GAG_dim_ D) with indication of the ion‐pair···π interactions. The C‐atoms of the peptide motifs are colored blue, and those of POPG_red_ and GAG_dim_ are colored green.

The interaction energies of the tetrapeptides with GAG_dim_ and POPG_red_ are presented in Figures 7 and 8. In general, these interaction energies are quite large due to the ion‐pair nature of the interactions, as GAG_dim_ and POPG_red_ are negatively charged, while the CPPs carry a positive charge. Since the solvent was simulated using a continuum model rather than explicit water molecules for computational efficiency, the absolute values of the interaction energies are likely overestimated. However, the relative energy differences between the different CPPs are more reliable. The interaction energies suggest that CPPs exhibit a slightly higher affinity for POPG compared to GAG, although the differences are relatively small. Notably, **2′** displays the lowest affinity within the series, which aligns well with experimental findings. Conversely, **11′** shows the highest affinity for both POPG and GAG. This trend is consistent with experimental results for GAG but deviates for POPG, where it exhibits the second‐highest affinity instead. Despite this minor discrepancy, the overall energetic analysis aligns with experimental observations, reinforcing the reliability of the DFT study even when using minimalistic models.

Figures 7 and 8 also illustrate the geometries of the optimized complexes, highlighting the ion‐pair interactions. In all cases, the side chains of the arginine residues engage in interactions with the anionic sulfate/carboxylate groups of GAGs or the phosphate group of POPG (indicated by dashed lines), forming charge‐assisted hydrogen bonds (salt bridges). These interactions are supported by the ion‐pair distances, which are in all cases shorter than 3.8 Å (C···C^ARG^ distance for carboxylate···ARG ion‐pair and O···C^ARG^ distance for sulfate/phosphate···ARG ion pairs). Some exemplifying distances are provided in the legend of Figure 7.

For CPPs containing aromatic rings, the ion‐pair interactions are positioned over the aromatic surfaces, exhibiting varying degrees of overlap. Upon examining the geometries, it is evident that **11′** displays the most effective ion‐pair···π contacts, where the cationic component of the ion‐pair is positioned closer to the aromatic surface than the anionic component. This observation aligns with the negative MEP values over the naphthalene ring, as revealed by the MEP surface analysis. Notably, in the **2′** complexes (Figure 8, top), the same ion‐pair contacts are established, with the key distinction being the absence of ion‐pair···π interactions compared to the other CPPs. Consequently, the difference in binding energy between the **2′**···POPG_red_ and **2′**···GAG_dim_ complexes and the other systems is primarily attributed to the ion‐pair···π interactions, which contribute between 5 and 12 kcal/mol. This underscores the significance of these interactions in stabilizing the complexes.

**Figure 9:**
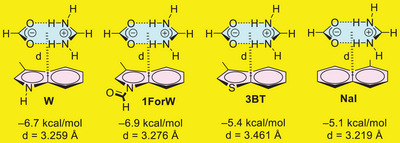
Model ion‐pair···π complexes analyzed in this work along with the interaction energies and interplane distances (d).

Supporting this interpretation, model ion‐pair systems involving formate–guanidinium pairs with W, 1ForW, 3BT, and NaI (Figure 9) were also computed, yielding interaction energies between −5.0 and −7.0 kcal/mol.

## 3 Discussion

Trp is a unique residue, which confers special properties to membrane proteins^[^ ^23^ ^]^ and membrane‐active peptides.^[^ ^24^ ^]^ Its importance for CPPs has been highlighted in numerous studies by substituting one or several Trp residues with other natural amino acids. The position and number of Trp in Arg/Trp sequences have also been extensively investigated. Increasing the number of Trp in Arg‐rich peptides is associated with better internalization faculties but can also lead to higher cytotoxicity.^[^ ^20^, ^21^ ^]^ The aim of the present study was to further investigate the role of Trp in the internalization mechanism. We systematically replaced all Trp residues in a very well characterized linear Arg‐rich CPP R_6_W_3_ by natural amino acids or nonnatural Trp analogues. We quantified their uptake in CHO cells and investigated their interactions with model lipid membranes and GAGs using calorimetry and DFT. Indeed, the routes through which CPPs enter cells are not yet fully understood, and our hypothesis is that the privileged membrane partners with which CPPs interact and the type of interaction they engage in are keys triggering endocytosis or direct translocation.^[^ ^27^ ^]^

Trp can engage in many different types of noncovalent interactions, and our objective was to delineate which type of interactions(s) was essential for efficient uptake. It appeared that 1/ H‐bond donation is not an important feature, 2/ substituents on the indole ring have little impac,t 3/ aromaticity is not sufficient to promote internalization, and 4/ hydrophobicity is important. Amongst the three most efficiently internalized R_6_X_3_ peptides were the ones carrying the most hydrophobic substituents, 3BT and Nal.

In linear Arg‐rich CPPs, the importance of large aromatic moieties to boost uptake of Arg‐rich peptides has long been highlighted, notably by the use of pyrene‐butyrate^[^ ^34^ ^]^ or other aromatic carboxylates as additive.^[^ ^35^ ^]^ It is also known that the addition of fluorophores such as rhodamine B or carboxyfluorescein,^[^ ^36^, ^37^ ^]^ which are large aromatic moieties, or the fluorescence quencher Dabcyl^[^ ^38^ ^]^ enhances CPP uptake. Arg‐rich cyclic peptides containing nonnatural aromatic acids have also been developed, notably the head‐to‐tail cyclized peptide CPP12 introduced by the Pei group in 2013 containing Pal^[^ ^39^ ^]^ and recently modified with 3BT, leading to better endosomal escape.^[^ ^40^ ^]^ Side‐chain macrocyclized Arg‐rich peptides with large aromatic staples have also enhanced uptake properties.^[^ ^41^ ^]^ Very interestingly, the effect of aromatic staples or substituents in cyclic and linear bioactive peptides has also been studied. These aromatic modifications had finely tuned hydrophobicity and electronic properties due to the presence of sulfur and fluorine atoms,^[^ ^42^ ^]^ and the combination of hydrophobicity and polarizability was key to efficient uptake of Arg‐rich CPPs, but also non‐penetrating sequences, and their presence promoted the interaction with lipid membranes.

Antimicrobial peptides (AMPs), another class of membrane‐active peptides, share common structural features with CPPs and also often contain Trp residues. Several studies involving Trp‐rich AMPs showed that substituting Trp residues with nonnatural aromatic and hydrophobic residues such as Nal or 3BT leads to increased antimicrobial properties, while less hydrophobic residues tend to decrease the activity.^[^ ^43^, ^44^, ^45^ ^]^

Seeing that hydrophobicity was a key feature for efficient internalization, we also first turned to the lipid membrane as a natural interaction partner. DSC revealed that all peptides in our small library interacted with negatively charged lipid headgroups, and peptides **9** and **6**, with 3BT and 1ForW substitutions, which carry permanently polarized aromatic side chains, led to the most striking disturbance of the main transition peak, a consequence of interactions with the hydrophobic part of the bilayer. Analogues with unsubstituted or substituted indole rings lead to the splitting of the main transition peak, which is often interpreted as lateral segregation in the bilayer. The existence of regions of different fluidity, and in particular the borders of such regions, has previously been proposed as possible entry routes for CPP direct translocation.^[^ ^46^ ^]^ and this lateral segregation could participate in the translocation mechanism of Arg/Trp peptides. Additionally, ITC showed that all peptides bound negatively charged membranes with µM affinity and that this binding was strongly entropy driven, but no obvious trend between binding and uptake in GAG‐deficient cells could be observed.

GAGs have long been identified as privileged partners for CPP binding at the cell surface and effectors of endocytosis and possibly direct translocation. Previously, we showed that increasing the number of Trp in Arg/Trp or Penetratin led to a more favorable binding enthalpy to GAGs and higher internalization in CHO cells.^[^ ^20^, ^26^ ^]^ Herein, we show that the highly internalized peptides **9** and **6** also exhibit larger binding enthalpies in interaction with HI, as well as the highest stoichiometries. Interactions between R_6_X_3_ peptides and HI surely result from a combination of electrostatic forces and H‐bonds involving guanidiniums of Arg and sulfates and/or carboxylates carried by HI. Additionally, aromatic side chains could engage in CH···π interactions with the carbohydrates of GAGs. In 2014, the group of Stefan Matile introduced a new type of interaction involving aromatics, which are ion‐pair···π interactions, a combination of anion···π and cation···π interactions occurring on the same aromatic surface.^[^ ^47^ ^]^ One year later, parallel ion‐pair···π interactions from anionic amphiphilic and aromatic activators have been shown to favor CPP insertion via translocation by making this entry pathway faster than endocytosis.^[^ ^48^ ^]^ This study involved two molecules, one carrying the aromatic surface and the negatively charged carboxylate group, the second corresponding to the CPP containing the positively charged guanidinium group of Arg. More recently, we suggested that this interaction could occur during Arg/Trp CPPs internalization, involving the guanidinium of Arg, a negatively charged moiety on the cell surface, namely a lipid phosphate, or a GAG carboxylate or sulfate, and finally the indole of Trp.^[^ ^20^ ^]^

The results of our DFT study further support the crucial role of ion‐pair···π interactions in the internalization mechanism of Arg‐rich CPPs. The computational analysis demonstrates that the presence of aromatic moieties, particularly those with extended hydrophobicity and polarizability, enhances the binding affinity of CPPs to both lipid membranes and GAGs. Specifically, our calculations reveal that the ion‐pair interactions between guanidinium groups of Arg residues and negatively charged sulfate or carboxylate groups in GAGs are further stabilized when an aromatic ring, such as Trp analogues, participates in the interaction. The presence of π‐systems contributes to the formation of strong ion‐pair···π interactions, effectively reinforcing peptide‐membrane and peptide‐GAG interactions. In particular, highly internalized peptides **6** and **9**, in which the aromatic systems are permanently polarized, also bind GAGs with the largest enthalpies. This aligns with previous experimental findings, which highlighted that aromatic substitutions enhance CPP uptake and endosomal escape. Furthermore, our energetic analysis shows that the contribution of ion‐pair···π interactions significantly differentiates highly internalized peptides from less efficient ones, reinforcing the idea that these interactions play a pivotal role in modulating CPP‐membrane interactions and facilitating cellular uptake.

## 4 Conclusion

CPPs internalization mechanism has widely been studied for over 35 years. Various amino acids seem to be essential in this mechanism, including tryptophan. However, the interactions in which it is involved are still not well understood. In this context, we substituted the three Trp residues in R_6_W_3_ sequences by other amino acids or analogs with different physicochemical properties. In our study, it appears that the combination of aromaticity/hydrophobicity in the presence of positively charged Arg within a peptide sequence favors its insertion in cell membranes and its internalization. We suggest that ion‐pair···π interactions at the origin of favorable binding enthalpy can be established between the extended aromatic ring, particularly permanently polarized ones, and the positively charged Arg forming an ion‐pair with negatively charged membrane partners such as GAGs or lipids. These interactions could contribute positively to CPP internalization, working in synergy with hydrophobic contact with the hydrophobic core of the lipid bilayer, providing new insight toward a better understanding of the mechanism of CPP internalization. These findings prove the involvement of such interactions in a biological context and can be applied to understand other biological processes.

## Supporting Information

Supporting information include Material and Methods, Table S1, Figures S1, S2 and S3, and cartesian coordinates of simulations.

## Conflict of Interest

The authors declare no conflict of interest.

## Supporting information



Supporting Information

## Data Availability

The data that support the findings of this study are available in the supplementary material of this article.
