# Supplementary material for: Hydrophobic and Polarized Aromatic Residues Promote Internalization of Arg‐Rich Cell‐Penetrating Peptides through Ionpair‐π Interactions
Source: Chemistry. 2025 Jul 4;31(41):e202501138. doi: 10.1002/chem.202501138 (PMC12284625; doi:10.1002/chem.202501138)
Supplement: Supplementary file 1 — Supporting Information [file CHEM-31-e202501138-s001.docx]

Hydrophobic And Polarized Aromatic Residues Promote Internalization Of Arg-Rich Cell-Penetrating Peptides Through Ionpair-π Interactions

Sonia Khemaissa^[a]^, Antonio Bauzá^[b]^, Émilie Lesur^[a],[c]^, Françoise Illien^[a]^, Sandrine Sagan^[a]^, Antonio Frontera^[b]^, Astrid Walrant*^[a]^

Supporting Information

**Material and Methods**

**Peptide synthesis**

MBHA Resin (0.53 mmol/g loading), HBTU, standard tert-butyloxycarbonyl (Boc) protected L-amino acids, Boc-L-2-Nal-OH, H-L-5-Fluoro-Trp-OH, H-L-5-MethoxyTrp-OH, Boc-L-Trp(For)-OH, were purchased from IrisBiotech GmbH. Boc-L-3-Pal was obtained from Bachem. Boc-Gly(D,D) was obtained from Eurisotop. H-3-(Thianaphthen-3-yl)-L-Ala and H-N-Me-Trp were obtained from Sigma Aldrich.

D-Biotin sulfone (Biot(O_2_) is obtained from oxidation of D-Biotin by 30% H_2_O_2_ in H_2_O treatment for 4 days and is used without further purification.

Trp analogues 5FW, 3BT, NMeW and 5OMeW were not Boc-protected. The protection of the α amine was performed using standard conditions with Boc_2_O as a protecting reagent.

All peptides were synthesized manually using Boc solid phase peptide synthesis, using a MBHA resin and HBTU as activation reagent. Side chains chain deprotection and peptide cleavage from the solid support was performed by anhydrous HF treatment.

Peptides were purified by RP-HPLC on a C18 preparative column (Macherey Nagel) and characterized by analytical RP-HPLC (ACE) and MALDI-TOF MS (positive ion linear mode).

Analytical RP HPLC was performed on a C18 analytical column (ACE), with a linear gradient 5% to 100% MeCN (0.1% TFA) over 10 min.

**Cell culture**

Wild type Chinese Hamster Ovary (CHO-K1, WT, ATCC) and xylose transferase-deficient CHO-pgsA745 (GAG-deficient, ATCC) were cultured in Dulbecco's modified Eagle's medium F-12 (DMEMF-12) supplemented with 10% fetal bovine serum (FBS), penicillin (100,000 IU/L), streptomycin (100,000 IU/L), and amphotericin B (1 mg/L) in a humidified atmosphere containing 5% CO_2_ at 37 °C.

**Internalization quantification by MS**

Quantification was performed as described previously ^[28]^.

Briefly, 500,000 cells/well were seeded in a 12-well plate the day prior to the experiment so that they reach 1,000,000 on the day of the experiment. The cells were incubated for 1h with 10 μM peptide at 37°C, then extensively washed with Hank’s Buffer Saline solution (HBSS). The cells were then treated with Trypsin, 5 min at 37°C to detach them from the wells and digest the non-internalized and membrane-bound peptide, and trypsin activity was stopped by addition of soybean trypsin inhibitor and bovine serum albumin. The cells were then harvested, the pellets washed and lysed (0.1% Triton X-100, 1M NaCl, 100°C, 15 min) in the presence of a known amount of deuterated peptide acting as an internal standard for MS quantification. The biotinylated peptides were retrieved by incubating the cell lysates with Dynabeads MyOne Streptavidin C1 (Invitrogen) 1h at room temperature. After several washing steps, the peptides were eluted from the beads by addition of CHCA matrix (saturated in H_2_O:MeCN 1/1, 0.1% TFA). Mass spectra were recorded by MALDI-TOF MS on a Voyager-DE Pro mass spectrometer (AppliedBiosystems) or Autoflex Speed 1000 Hz (Bruker) in positive ion linear mode. To ensure good representativeness of the sample, thousands of shots were accumulated several times for each deposit.

**Cytotoxicity assays**

7,000 cells/well were seeded in a 96-well plate the day prior to the experiment to reach 14,000 on the day of the experiment. For cytotoxicity assays, the Cell Counting Kit-8 (Dojindo) was been used. Cells were incubated with three different peptide concentrations 10, 25 (for **9** only), 50 μM for 1h at 37°C. The experiments have been performed in triplicate. 3 wells were used as a positive control containing only DMEM-F12 medium and 3 wells were used as a negative control containing 0.1% Triton X-100 treatment in medium.

**Calorimetry experiments**

*Preparation of MLVs and LUVs lipid films*

MLVs were obtained using the standard dried lipid film method and were resuspended in PBS. MLVs were directly used for DSC experiments.

For ITC experiments, LUVs were prepared from MLVs after five freeze-thaw cycles in liquid N_2_/warm water before extruding them 15 times through a 100 nm polycarbonate membrane by a mini-extruder (Avanti) to obtain uniformly sized liposomes.

*Isothermal titration calorimetry (ITC)*

ITC experiments were performed on a TA Instruments nano ITC calorimeter. Heparin or POPG LUVs were introduced in a 250 μL injection syringe with concentrations in PBS ranging between 8 and 10 μM for heparin and 1.5 and 5 mM total lipid for POPG LUVs. Heparin was obtained as a concentrated stock solution (25.000 UI/ 5 mL, 3 mM) from Sanofi. The peptide solution (concentration between 30 μM and 75 μM in PBS) was placed in a 983 μL measuring cell. The experiment was performed at 25°C. Heparin or POPG LUVs were injected by steps (1 × 2 μL followed by 24 × 10 μL) every 5 minutes into the measuring cell. The experiments were performed twice.

Data analysis was performed using NanoAnalyze software provided by TA instruments. A simple model with n independent binding sites was used to determine the binding parameters.

*Differential scanning calorimetry (DSC)*

DSC experiments were performed on a high-sensitivity micro DSC calorimeter (TA Instruments). MLVs of DMPG at 1 mg/mL in PBS were introduced in the measuring cell. The experiments were conducted at an overpressure of 3 atm. Five heating/cooling cycles from 0°C to 50°C were applied with a scan rate of 1°C/min. The peptide was gradually added to reach peptide/lipid ratios of 1/100, 1/50 and 1/25. Data analysis was performed using NanoAnalyze software provided by TA instruments.

**Surface Pressure measurements**

Monolayer experiments were performed in a Langmuir-Blodgett Teflon trough (KSV NIMA, 98 cm^2^ surface area, 57 mL volume). The surface pressure was measured using the Wilhelmly method using a platinium plate. All the experiments were performed at room temperature. The trough was filled with PBS, pH 7.4. Monolayers were obtained by depositing 15 μL of a DMPC/DMPG (80:20) solution in CHCl_3_ (0,5 mg/mL total lipid) at the air/buffer interface. The monolayer was then compressed to reach 27 mN/m. 10 μL of each peptide in solution (1 mM) was then injected under the monolayer into the subphase and the surface pressure was monitored continuously.

**Theoretical methods**

The energies of all complexes studied in this work were computed at the BP86-D3/def2-TZVP level of theory ^[49-52]^ using the TURBOMOLE version 7.2 program ^[53]^. No constraints were imposed during the optimization procedures. The minimum-energy character of the complexes was confirmed through frequency calculations conducted at the same level of theory. Interaction energies were determined by subtracting the sum of the energies of the individually optimized monomers from the energy of the fully optimized assembly. MEP surfaces were calculated at the same theoretical level and visualized on the van der Waals isosurface (0.001 a.u.). To incorporate solvent effects, the conductor-like screening model (COSMO) ^[54,55]^, a variant of dielectric continuum solvation models, was employed, with water chosen as the solvent. We acknowledge that the presence of explicit water molecules would likely reduce the calculated binding energies reported in the manuscript, as water could compete with some of the hydrogen bonds observed in the optimized geometries using a continuum model. However, our primary focus is on the structural features and binding interactions relevant once the CPPs are embedded within or interacting with the membrane environment—where water accessibility is significantly reduced. In this context, the continuum model is a reasonable compromise that captures key interaction patterns.

In addition, the interaction energies of the four supramolecular complexes involving a carboxylate···guanidinium ion pair and the different π-systems used were also computed at the BP86-D3/def2-TZVP level of theory. More in detail, the distance between the ion pair and the π-system was scanned to find a local noncovalent minimum based on a ion pair-π interaction at the BP86-D3/def2-SVP level of theory. To achieve this, we scanned the distance between the two moieties from 2.4 to 4.0Å using a stepsize of 0.2Å. Once a noncovalent minima was found, single point calculations were performed using the def2-TZVP basis set and the COSMO approach to obtain the interaction energy values.

**Table S1.** Peptide library. The observed [M+H]^+^ were obtained by MALDI-TOF MS in reflector positive ion mode, CHCA matrix. For deuterated peptides, the first observed isotope corresponds to peptides with not fully deuterated Gly.

**Figure S1.** Cytotoxicity of R_6_W_3_ peptides on CHO-K1 cells determined by the CCK8 cytotoxicity assay for 10 and 50 μM extracelullar concentrations (and 25 μM for **9**). Experiments were performed twice in triplicate and error bars show SDs.

**Figure S2.** Enthalpy/entropy compensation for peptides interacting with HI studied by ITC.

**Figure S3.** Variation of the surface pressure after injection of peptides **2** and **6** in the subsphase of a DMPC/DMPG (80/20) monolayer. The black arrow indicates the time of injection.

**Cartesian coordinates**

**Ion pair@W**

(ΔE_vaccum_ = –6.7 kcal/mol, ΔE_water_ = –1.5 kcal/mol)

| C | 2.62539800 | -1.32291000 | 0.00242900 |
| --- | --- | --- | --- |
| C | 1.60795300 | -0.22682900 | -0.00140500 |
| C | 1.84025800 | 1.13102200 | 0.00058900 |
| C | 0.17749900 | -0.39155000 | -0.00311100 |
| N | 0.63489800 | 1.81769700 | -0.00148400 |
| C | -0.40518000 | 0.91148600 | -0.00184800 |
| C | -0.66955400 | -1.51405900 | -0.00313400 |
| C | -1.78987400 | 1.11065100 | -0.00016200 |
| C | -2.04775900 | -1.32003300 | 0.00107600 |
| C | -2.60147000 | -0.02176600 | 0.00283700 |
| H | 2.52167000 | -1.96367100 | 0.89240200 |
| H | 3.64730800 | -0.92078300 | -0.00438400 |
| H | 2.51531500 | -1.97692200 | -0.87703100 |
| H | 2.78298900 | 1.67031700 | 0.00494100 |
| H | 0.53533100 | 2.82383000 | 0.00769500 |
| H | -0.24933200 | -2.52141000 | -0.00490600 |
| H | -2.22018000 | 2.11359600 | -0.00238900 |
| H | -2.71548200 | -2.18257400 | 0.00317700 |
| H | -3.68552900 | 0.09767100 | 0.00725500 |
| C | 1.45136504 | 1.11069145 | 3.18917004 |
| O | 0.45700904 | 1.89576645 | 3.19307304 |
| O | 1.44304404 | -0.15643955 | 3.18808004 |
| H | 2.45705204 | 1.59376845 | 3.18634804 |
| C | -1.94921396 | -0.52290855 | 3.19871204 |
| N | -0.87891896 | -1.28973255 | 3.19457904 |
| N | -1.87924796 | 0.79182345 | 3.19964404 |
| H | -2.93788496 | -0.99799955 | 3.20148604 |
| H | 0.14190004 | -0.82724655 | 3.19169004 |
| H | -1.00947396 | -2.29432055 | 3.19411604 |
| H | -0.88039196 | 1.29956245 | 3.19686604 |
| H | -2.74516196 | 1.31757445 | 3.20290404 |

**Ion pair@ForW**

(ΔE_vaccum_ = –6.9 kcal/mol, ΔE_water_ = –3.3 kcal/mol)

| C | -0.12328600 | 3.11652900 | 0.00054200 |
| --- | --- | --- | --- |
| C | 0.17815900 | 1.65431900 | -0.00088100 |
| C | 1.40780400 | 1.05931700 | -0.00285800 |
| C | -0.80688700 | 0.58926300 | 0.00071800 |
| C | -0.10843400 | -0.64532700 | -0.00056000 |
| C | -2.20843000 | 0.58864100 | 0.00161000 |
| C | -0.77118100 | -1.87259400 | -0.00154700 |
| C | -2.87886000 | -0.63401800 | 0.00102800 |
| C | -2.16823400 | -1.84733600 | -0.00038800 |
| N | 1.26653500 | -0.33811500 | -0.00229200 |
| C | 2.32620800 | -1.23581200 | 0.00113500 |
| O | 3.49753400 | -0.90469100 | 0.00280400 |
| H | -0.72828700 | 3.39593800 | -0.87615600 |
| H | 0.79557500 | 3.71624900 | -0.01405200 |
| H | -0.70245100 | 3.39946000 | 0.89334100 |
| H | 2.40581500 | 1.48381000 | -0.00463500 |
| H | -2.76138700 | 1.52911500 | 0.00252300 |
| H | -0.23713800 | -2.82218800 | -0.00374800 |
| H | -3.96918100 | -0.65350700 | 0.00175200 |
| H | -2.71541700 | -2.79043400 | -0.00058400 |
| H | 1.98529800 | -2.29199500 | 0.00237700 |
| C | -2.11127482 | -0.97492838 | 3.17026760 |
| O | -1.06178782 | -1.68462838 | 3.17224260 |
| O | -2.19641182 | 0.28927262 | 3.18574160 |
| H | -3.07855182 | -1.53066838 | 3.15277060 |
| C | 1.15941418 | 0.90437062 | 3.22943160 |
| N | 0.03554418 | 1.59028262 | 3.22635560 |
| N | 1.18658718 | -0.41183438 | 3.21266660 |
| H | 2.11031118 | 1.45089562 | 3.24663460 |
| H | -0.94834082 | 1.05396262 | 3.20893360 |
| H | 0.09166118 | 2.60167062 | 3.24005760 |
| H | 0.22794118 | -0.99166538 | 3.19494960 |
| H | 2.08886718 | -0.87238738 | 3.21630360 |

**Ion pair@3BT**

(ΔE_vaccum_ = –5.4 kcal/mol, ΔE_water_ = –2.8 kcal/mol)

| C | 2.10662800 | 2.06368200 | -0.00101400 |
| --- | --- | --- | --- |
| C | 1.42216500 | 0.72987900 | 0.00012600 |
| C | 2.05958200 | -0.48038200 | -0.00039900 |
| C | -0.01206900 | 0.57080200 | 0.00119100 |
| C | -0.40774300 | -0.79533200 | 0.00075400 |
| C | -1.01174000 | 1.56303700 | 0.00139100 |
| C | -1.75676800 | -1.17009000 | -0.00075800 |
| C | -2.35144500 | 1.19179400 | -0.00004600 |
| C | -2.72217600 | -0.16693400 | -0.00105200 |
| S | 0.98387000 | -1.84779800 | 0.00042200 |
| H | 1.81387800 | 2.65730300 | -0.88121700 |
| H | 3.19846800 | 1.95444800 | -0.01271000 |
| H | 1.83198300 | 2.65018100 | 0.88973100 |
| H | 3.13360900 | -0.65185700 | -0.00208800 |
| H | -0.72945400 | 2.61731200 | 0.00246100 |
| H | -2.04459300 | -2.22185700 | -0.00187500 |
| H | -3.12617400 | 1.95946300 | -0.00025900 |
| H | -3.77824100 | -0.43898000 | -0.00195100 |
| C | -1.78874880 | 0.26961996 | 3.38149228 |
| O | -1.56408580 | -0.97715704 | 3.36836728 |
| O | -0.94302380 | 1.21308896 | 3.39903728 |
| H | -2.86206680 | 0.57415996 | 3.37706328 |
| C | 1.84061520 | -0.76002304 | 3.39646728 |
| N | 1.54698220 | 0.52340096 | 3.40951028 |
| N | 0.91718120 | -1.69829704 | 3.37840128 |
| H | 2.89589920 | -1.05926904 | 3.40082328 |
| H | 0.47597020 | 0.85338696 | 3.40540128 |
| H | 2.31032320 | 1.18925796 | 3.42314128 |
| H | -0.16735980 | -1.41675404 | 3.37361628 |
| H | 1.21744620 | -2.66576204 | 3.36916128 |

**Ion pair@NaI**

(ΔE_vaccum_ = –5.1 kcal/mol, ΔE_water_ = –1.9 kcal/mol)

| C | 1.84058600 | -1.72192500 | -0.00076100 |
| --- | --- | --- | --- |
| C | 0.49199800 | -2.00007200 | 0.00057200 |
| C | -0.45823900 | -0.94428000 | 0.00096600 |
| C | 0.00149000 | 0.41953300 | 0.00007500 |
| C | 1.40980700 | 0.68639000 | -0.00027900 |
| C | 2.29140300 | -0.38102000 | -0.00098700 |
| C | -1.85481100 | -1.20322200 | 0.00155000 |
| C | -0.97251400 | 1.45547600 | -0.00086200 |
| C | -2.32353800 | 1.16913200 | -0.00102200 |
| C | -2.77101600 | -0.17217600 | 0.00036600 |
| C | 1.92500400 | 2.10256400 | 0.00046900 |
| H | 2.56996600 | -2.53332100 | -0.00139800 |
| H | 0.13478500 | -3.03191900 | 0.00097400 |
| H | 3.36557600 | -0.18302100 | -0.00155900 |
| H | -2.19240200 | -2.24182600 | 0.00262800 |
| H | -0.64813600 | 2.49627200 | -0.00190500 |
| H | -3.05150500 | 1.98185700 | -0.00195500 |
| H | -3.84036400 | -0.38760900 | 0.00045200 |
| H | 1.57914500 | 2.65969800 | -0.88413100 |
| H | 1.57964200 | 2.65821100 | 0.88619200 |
| H | 3.02227900 | 2.11926200 | 0.00017700 |
| C | -1.86767375 | 0.02039266 | 3.17653496 |
| O | -1.71464575 | -1.23725434 | 3.17413096 |
| O | -0.96936975 | 0.91410966 | 3.18015396 |
| H | -2.92186175 | 0.38571966 | 3.17531896 |
| C | 1.69700825 | -1.21479834 | 3.18064796 |
| N | 1.47721425 | 0.08337466 | 3.18301296 |
| N | 0.72141725 | -2.09889334 | 3.17690396 |
| H | 2.73349325 | -1.57381034 | 3.18184496 |
| H | 0.42678525 | 0.47397666 | 3.18185796 |
| H | 2.27739625 | 0.70461766 | 3.18586096 |
| H | -0.34528975 | -1.75588634 | 3.17561596 |
| H | 0.96589325 | -3.08198034 | 3.17526096 |

**RQQR@POPG**

| N | -3.2397128 | -7.2828967 | 0.9508491 |
| --- | --- | --- | --- |
| H | -3.2379261 | -6.7507913 | 1.8239454 |
| C | -3.3067109 | -6.3494143 | -0.1743666 |
| H | -3.3165299 | -6.9529802 | -1.0986366 |
| C | -4.5040189 | -5.3806376 | -0.1846340 |
| C | -2.0224966 | -5.5175326 | -0.1286441 |
| H | -5.4398913 | -5.9553405 | -0.2361419 |
| H | -4.5138553 | -4.8664154 | 0.7898939 |
| C | -4.4511481 | -4.3540894 | -1.3296182 |
| O | -1.8813724 | -4.5965227 | 0.7029089 |
| H | -3.4149375 | -4.0475500 | -1.5443702 |
| H | -4.8129135 | -4.8216832 | -2.2568166 |
| C | -5.2746995 | -3.0779242 | -1.0371281 |
| H | -5.9380135 | -2.8446879 | -1.8784208 |
| H | -5.9181354 | -3.2348887 | -0.1575715 |
| N | -4.4900821 | -1.8582524 | -0.8156523 |
| H | -4.4943387 | -1.1287189 | -1.5405091 |
| C | -3.9095531 | -1.4874431 | 0.3367447 |
| N | -3.5854726 | -2.3897243 | 1.2902442 |
| N | -3.6734048 | -0.1944544 | 0.5685388 |
| H | -3.1677262 | -3.2924160 | 1.0103585 |
| H | -3.1681370 | -1.9799501 | 2.1253786 |
| H | -4.0354865 | 0.4878462 | -0.0905509 |
| H | -2.9636164 | 0.0568044 | 1.2921012 |
| N | -1.0576660 | -5.8265574 | -1.0151186 |
| H | -1.2718973 | -6.4967261 | -1.7468855 |
| C | 0.1225999 | -4.9923805 | -1.1799330 |
| H | 0.5575949 | -4.8252553 | -0.1861394 |
| C | 1.1458166 | -5.6950341 | -2.0873648 |
| C | -0.2985417 | -3.6437763 | -1.7986100 |
| H | 1.1925309 | -6.7563086 | -1.7977383 |
| H | 0.8056260 | -5.6452249 | -3.1329152 |
| O | -1.1384515 | -3.5818270 | -2.7003364 |
| N | 0.3632075 | -2.5633935 | -1.3237303 |
| H | 0.9805067 | -2.6466929 | -0.5134639 |
| C | 0.1931906 | -1.2666274 | -1.9459480 |
| H | 0.2716770 | -1.4239681 | -3.0381069 |
| C | -1.1944037 | -0.6365122 | -1.6597385 |
| C | 1.3891525 | -0.3857229 | -1.5401478 |
| H | -1.8870010 | -1.4569838 | -1.4471693 |
| H | -1.1342949 | -0.0247464 | -0.7491504 |
| O | 2.4519870 | -0.8553177 | -1.1246064 |
| N | 1.2025725 | 0.9440920 | -1.7267706 |
| H | 0.2781828 | 1.2998299 | -1.9490338 |
| C | 2.1493199 | 1.9486547 | -1.2882932 |
| H | 2.3758190 | 1.7919907 | -0.2200789 |
| C | 3.4809074 | 1.9307636 | -2.0951591 |
| C | 1.4352844 | 3.2893462 | -1.4103743 |
| H | 3.5743173 | 0.9274482 | -2.5306254 |
| H | 3.4034753 | 2.6349819 | -2.9362747 |
| C | 4.7424484 | 2.2423411 | -1.2786633 |
| O | 0.2924562 | 3.4280047 | -1.8267718 |
| H | 5.5682743 | 2.4231809 | -1.9822073 |
| H | 4.6189552 | 3.1788237 | -0.7112814 |
| C | 5.1956836 | 1.1156016 | -0.3357127 |
| H | 5.0887005 | 0.1572597 | -0.8626481 |
| H | 6.2615680 | 1.2426170 | -0.0874746 |
| N | 4.4171467 | 1.0725670 | 0.9000786 |
| H | 4.1877172 | 1.9680255 | 1.3406302 |
| C | 3.9182723 | -0.0136354 | 1.5089329 |
| N | 4.2095159 | -1.2678596 | 1.1184823 |
| N | 3.1471578 | 0.1613812 | 2.5966128 |
| H | 4.6573591 | -1.3616362 | 0.2134676 |
| H | 3.4212153 | -1.9317333 | 1.2523272 |
| H | 2.6593529 | 1.0492465 | 2.7171619 |
| H | 2.6667473 | -0.6457126 | 2.9805002 |
| C | -4.2852735 | -8.3068551 | 0.9685461 |
| H | -4.1266423 | -8.9618947 | 1.8350123 |
| H | -5.3208930 | -7.9181291 | 1.0166821 |
| H | -4.1983303 | -8.9275014 | 0.0645643 |
| O | 2.2132759 | 4.3062959 | -1.0199167 |
| C | 1.6217507 | 5.6338145 | -1.1370811 |
| H | 2.4239465 | 6.3265623 | -0.8690842 |
| H | 1.2830242 | 5.8063285 | -2.1653148 |
| H | 0.7777103 | 5.7221814 | -0.4419674 |
| O | 2.4984191 | -5.1010026 | 1.6197658 |
| H | 2.6267399 | -4.4124572 | 0.9396560 |
| C | 1.3249866 | -4.7097252 | 2.3348588 |
| H | 0.4052686 | -4.9721683 | 1.7784531 |
| H | 1.3219128 | -5.2675394 | 3.2812595 |
| C | 1.3294607 | -3.2041390 | 2.6129771 |
| H | 2.0793373 | -2.9816415 | 3.3958773 |
| O | 1.7316835 | -2.5331592 | 1.3966052 |
| H | 1.1911321 | -1.6607842 | 1.2900852 |
| C | -0.0336469 | -2.7247879 | 3.0843848 |
| H | -0.3749230 | -3.3323203 | 3.9347278 |
| H | -0.7613703 | -2.8317605 | 2.2626501 |
| O | 0.0414214 | -1.3588886 | 3.5614402 |
| P | -0.3859917 | -0.1656809 | 2.5478316 |
| O | -1.8701200 | 0.1077403 | 2.5493708 |
| O | 0.2830612 | -0.4066740 | 1.1998321 |
| O | 0.4009989 | 1.0153185 | 3.3507291 |
| C | -0.0828770 | 2.3643118 | 3.2700012 |
| H | -1.1358469 | 2.4063083 | 3.5823407 |
| H | 0.5161462 | 2.9424208 | 3.9836010 |
| C | 0.0816334 | 2.9468762 | 1.8475842 |
| H | 0.6406448 | 2.2153555 | 1.2437290 |
| C | -1.2415912 | 3.2167336 | 1.1659224 |
| H | -1.0847430 | 3.6104287 | 0.1506706 |
| H | -1.7530868 | 2.2452023 | 1.1026683 |
| O | 0.8346081 | 4.2040915 | 1.8682405 |
| C | 2.1655155 | 4.1002695 | 2.0212318 |
| C | 2.8514113 | 5.4365523 | 2.1067577 |
| H | 2.2105943 | 6.2046602 | 1.6536400 |
| H | 2.9068461 | 5.6857953 | 3.1809790 |
| C | 4.2575692 | 5.4239440 | 1.4992456 |
| H | 4.8635023 | 4.6605331 | 2.0119299 |
| H | 4.1888545 | 5.1080429 | 0.4454792 |
| C | 4.9537445 | 6.7841488 | 1.5847929 |
| H | 5.0297092 | 7.0929824 | 2.6412923 |
| H | 4.3313127 | 7.5508969 | 1.0904540 |
| C | 6.3491045 | 6.7794091 | 0.9522127 |
| H | 6.9660865 | 6.0097617 | 1.4462171 |
| H | 6.2652661 | 6.4718561 | -0.1043730 |
| C | 7.0500848 | 8.1371172 | 1.0402960 |
| H | 7.1791717 | 8.4490200 | 2.0871875 |
| H | 8.0449685 | 8.1059145 | 0.5757512 |
| H | 6.4673524 | 8.9189345 | 0.5305998 |
| O | 2.7449312 | 3.0194188 | 2.1012484 |
| O | -1.9747343 | 4.1749313 | 1.9458553 |
| C | -3.3567563 | 4.2133048 | 1.9355772 |
| O | -3.8973933 | 5.1225649 | 2.5144409 |
| C | -4.0828502 | 3.0806397 | 1.2374134 |
| H | -3.6312904 | 2.9039844 | 0.2466586 |
| H | -3.8841028 | 2.1665375 | 1.8242240 |
| C | -5.5866940 | 3.3120425 | 1.1000543 |
| H | -5.7594857 | 4.2449663 | 0.5404546 |
| H | -6.0199719 | 3.4784900 | 2.0971719 |
| C | -6.2835891 | 2.1401141 | 0.4031205 |
| H | -5.8108131 | 1.9540645 | -0.5808851 |
| H | -6.1275286 | 1.2199121 | 0.9937999 |
| C | -7.7857879 | 2.3537254 | 0.1973567 |
| H | -7.9443626 | 3.2706546 | -0.3941959 |
| H | -8.2625664 | 2.5363275 | 1.1747723 |
| C | -8.4596310 | 1.1675523 | -0.4963403 |
| H | -9.5356809 | 1.3386791 | -0.6372212 |
| H | -8.0165030 | 0.9857339 | -1.4876482 |
| H | -8.3435405 | 0.2460197 | 0.0947840 |
| C | -1.7449340 | 0.1545974 | -2.8683813 |
| H | -0.9618538 | 0.7568526 | -3.3557367 |
| H | -2.1067871 | -0.5584186 | -3.6222484 |
| C | 2.5479466 | -5.0862963 | -1.9826962 |
| H | 2.4956928 | -3.9889571 | -2.0705934 |
| H | 2.9859524 | -5.3241750 | -1.0009137 |
| C | 3.4377553 | -5.5607853 | -3.1336687 |
| C | -2.9067519 | 1.0433000 | -2.4683344 |
| O | 3.0744324 | -5.4746426 | -4.3013959 |
| O | -4.0436071 | 0.5929006 | -2.2457266 |
| N | 4.6657006 | -6.0438688 | -2.7743448 |
| H | 5.2646468 | -6.4132447 | -3.5062045 |
| H | 4.9047279 | -6.2394404 | -1.8100842 |
| N | -2.6340805 | 2.3625860 | -2.3145648 |
| H | -3.3987395 | 2.9890619 | -2.0820592 |
| H | -1.7092654 | 2.7584501 | -2.4634418 |

**RQQR@GAG**

| N | -3.7565333 | -6.6810007 | -0.0442361 |
| --- | --- | --- | --- |
| H | -3.5226561 | -6.5653773 | 0.9449770 |
| C | -3.5554384 | -5.3980423 | -0.7139066 |
| H | -3.6149739 | -5.5784903 | -1.8026883 |
| C | -4.5589101 | -4.2747251 | -0.3516289 |
| C | -2.1492045 | -4.9174948 | -0.3515566 |
| H | -5.5281173 | -4.5071105 | -0.8218175 |
| H | -4.7188986 | -4.2993120 | 0.7391412 |
| C | -4.1010744 | -2.8661088 | -0.7510395 |
| O | -1.8345681 | -4.7093052 | 0.8257337 |
| H | -3.2991863 | -2.5400486 | -0.0693525 |
| H | -3.6719672 | -2.8519747 | -1.7663257 |
| C | -5.2650894 | -1.8670973 | -0.6598619 |
| H | -5.9013110 | -1.9445091 | -1.5541609 |
| H | -5.9057123 | -2.1166039 | 0.1990314 |
| N | -4.8589610 | -0.4775888 | -0.5188725 |
| H | -4.5896293 | 0.0765045 | -1.3415297 |
| C | -4.7711383 | 0.1590595 | 0.6645431 |
| N | -4.9012178 | -0.5374681 | 1.8225557 |
| N | -4.6227810 | 1.4817170 | 0.6853552 |
| H | -4.4421118 | -1.4561627 | 1.8442862 |
| H | -4.5941563 | -0.0128431 | 2.6477114 |
| H | -4.5130629 | 1.9743706 | -0.2034537 |
| H | -4.2898954 | 1.9836335 | 1.5244401 |
| N | -1.3121290 | -4.7302157 | -1.4061022 |
| H | -1.7341494 | -4.7370722 | -2.3326603 |
| C | -0.0571891 | -3.9999779 | -1.3293000 |
| H | 0.2473798 | -3.9333914 | -0.2766436 |
| C | 1.0345619 | -4.7256466 | -2.1539019 |
| C | -0.2922440 | -2.6042269 | -1.9432319 |
| H | 1.0706587 | -5.7699966 | -1.8071868 |
| H | 0.7144288 | -4.7289392 | -3.2095193 |
| O | -1.0357280 | -2.4874545 | -2.9309468 |
| N | 0.3961372 | -1.5730984 | -1.4059787 |
| H | 0.9571875 | -1.7044289 | -0.5375149 |
| C | 0.2925558 | -0.2808827 | -2.0511045 |
| H | 0.2791679 | -0.4705130 | -3.1384653 |
| C | -1.0327166 | 0.4595331 | -1.6925357 |
| C | 1.5004596 | 0.5988527 | -1.7095379 |
| H | -1.7476258 | -0.3003161 | -1.3495344 |
| H | -0.8713051 | 1.1302307 | -0.8380728 |
| O | 2.2006720 | 0.4626040 | -0.7057408 |
| N | 1.7161498 | 1.6079165 | -2.6111001 |
| H | 1.0382617 | 1.7207716 | -3.3619660 |
| C | 2.4511717 | 2.8157419 | -2.2660340 |
| H | 2.4534275 | 2.8947900 | -1.1628242 |
| C | 3.9266688 | 2.8539226 | -2.7286065 |
| C | 1.6213594 | 4.0122762 | -2.7441647 |
| H | 3.9916213 | 2.4596910 | -3.7548952 |
| H | 4.2331574 | 3.9064409 | -2.7714969 |
| C | 4.8756027 | 2.1030996 | -1.7763645 |
| O | 0.5040627 | 3.9231312 | -3.2314570 |
| H | 5.8531412 | 2.6116327 | -1.7672118 |
| H | 4.4715930 | 2.1726061 | -0.7535171 |
| C | 5.1022663 | 0.6182178 | -2.0946908 |
| H | 4.1484332 | 0.1126373 | -2.2823744 |
| H | 5.7286554 | 0.4993617 | -2.9913543 |
| N | 5.7333554 | -0.0484293 | -0.9416459 |
| H | 6.4012468 | 0.5282394 | -0.4343711 |
| C | 5.0036626 | -0.8896529 | -0.1452092 |
| N | 4.3047828 | -1.8709434 | -0.6891585 |
| N | 5.0329813 | -0.7408500 | 1.1879452 |
| H | 4.4994723 | -2.2287514 | -1.6339557 |
| H | 3.4989025 | -2.2696995 | -0.1427839 |
| H | 5.1879908 | 0.1882266 | 1.5664426 |
| H | 4.4517137 | -1.3975245 | 1.7722370 |
| C | -5.0847241 | -7.2637426 | -0.2095156 |
| H | -5.1087285 | -8.2431372 | 0.2869944 |
| H | -5.9204102 | -6.6564867 | 0.1920479 |
| H | -5.2771313 | -7.4299778 | -1.2810993 |
| O | 2.2484055 | 5.1736267 | -2.5175807 |
| C | 1.4989353 | 6.3709666 | -2.8689573 |
| H | 2.1404519 | 7.2057437 | -2.5749173 |
| H | 1.2996287 | 6.3849100 | -3.9476353 |
| H | 0.5505771 | 6.3957570 | -2.3186368 |
| C | -0.4704855 | 3.8525583 | 0.9554861 |
| C | 0.3281040 | 2.5706395 | 1.2078025 |
| C | 1.4289087 | 2.7912514 | 2.2547467 |
| C | 2.2450952 | 4.0337265 | 1.8133125 |
| C | 0.4435116 | 5.0418388 | 0.6625937 |
| H | 0.8338536 | 2.2919577 | 0.2631243 |
| H | 1.0088123 | 3.0081799 | 3.2477306 |
| H | 2.7280142 | 3.8134779 | 0.8195454 |
| C | 0.8285187 | -1.4876484 | 2.4476895 |
| C | -0.4375971 | 0.4655628 | 2.2606832 |
| C | -1.6562615 | -1.7006814 | 2.9010017 |
| C | -0.3780592 | -2.4232545 | 2.4349412 |
| H | -0.0442671 | 0.7519524 | 3.2572115 |
| H | -1.6574991 | -1.6462320 | 4.0041701 |
| H | -0.5249271 | -2.7270270 | 1.3877535 |
| H | 0.9386746 | 4.8259079 | -0.3136847 |
| H | 1.0549759 | -1.1178966 | 3.4625580 |
| O | -0.6821675 | 1.5811275 | 1.4616251 |
| O | 1.4368443 | 5.1916218 | 1.6717666 |
| C | -1.7881428 | -0.2509570 | 2.3928323 |
| H | -2.1989106 | -0.2932861 | 1.3639485 |
| O | 0.4943506 | -0.3814628 | 1.5922974 |
| O | -0.1839312 | -3.6502620 | 3.1338911 |
| O | -2.8154621 | -2.4556241 | 2.4812332 |
| H | -2.5087479 | -3.3387247 | 2.1765314 |
| O | -2.6489924 | 0.4568961 | 3.2800836 |
| H | -2.6650667 | 1.4197708 | 3.0407858 |
| H | -1.1226317 | 3.6776563 | 0.0865617 |
| O | -1.2699349 | 4.1171981 | 2.1069256 |
| O | 3.2159687 | 4.2722402 | 2.7718836 |
| C | -0.2950547 | 6.3847852 | 0.4866871 |
| H | 0.4232443 | 7.0916741 | 0.0403690 |
| H | -1.1038173 | 6.2077147 | -0.2546039 |
| O | -0.7621712 | 7.0061552 | 1.6569962 |
| H | -1.5266073 | 6.5087471 | 2.0234711 |
| C | 4.1518070 | 5.2862010 | 2.3977589 |
| H | 4.9072044 | 5.3162231 | 3.1913712 |
| H | 4.6400053 | 5.0344787 | 1.4358079 |
| H | 3.6656370 | 6.2689117 | 2.3088540 |
| C | 0.3190239 | -3.5346141 | 4.4689086 |
| H | -0.2739909 | -2.8369842 | 5.0862959 |
| H | 0.2419425 | -4.5372239 | 4.9067472 |
| H | 1.3753037 | -3.2171680 | 4.4804036 |
| C | 2.1087928 | -2.1078073 | 1.8611639 |
| O | 2.0310143 | -2.5150249 | 0.6552432 |
| O | 3.1437412 | -2.1042897 | 2.5751063 |
| S | -2.9248335 | 4.2306071 | 1.9511000 |
| O | -3.1954718 | 5.5163736 | 2.5787947 |
| O | -3.2231112 | 4.1481943 | 0.5091962 |
| O | -3.4332520 | 3.0553395 | 2.7010679 |
| C | 2.6876555 | 1.1221977 | 3.5729467 |
| O | 2.4139367 | 1.6045761 | 4.6636758 |
| N | 2.3211492 | 1.6458299 | 2.3605550 |
| H | 3.2573624 | 0.1732410 | 3.4559034 |
| H | 2.3807287 | 1.0258301 | 1.5516222 |
| C | 2.3900581 | -4.0356944 | -1.9974981 |
| H | 2.2591665 | -2.9501967 | -2.0868974 |
| H | 2.7625957 | -4.1966924 | -0.9740277 |
| C | -1.6551646 | 1.2103554 | -2.8865807 |
| H | -0.9080180 | 1.8178866 | -3.4169246 |
| H | -2.0800989 | 0.4845365 | -3.5925919 |
| C | 3.5292258 | -4.3606599 | -2.9420816 |
| C | -2.7516389 | 2.1165123 | -2.3612133 |
| O | 4.4884821 | -3.5831551 | -3.0552927 |
| N | 3.4692431 | -5.5242886 | -3.6392530 |
| H | 4.2463381 | -5.7709394 | -4.2428951 |
| H | 2.7031190 | -6.1770892 | -3.5313237 |
| O | -3.8764641 | 1.6711300 | -2.0666026 |
| N | -2.4101605 | 3.4049712 | -2.1597635 |
| H | -2.9726396 | 3.9651579 | -1.5152416 |
| H | -1.4749291 | 3.7258087 | -2.3970065 |

**RforWForWR@POPG**

| N | -2.5368595 | -7.6595223 | 0.4845410 |
| --- | --- | --- | --- |
| H | -2.7657213 | -7.1697518 | 1.3524534 |
| C | -2.4337882 | -6.6897868 | -0.6074265 |
| H | -2.2220014 | -7.2635397 | -1.5269450 |
| C | -3.6658553 | -5.7991518 | -0.8508368 |
| C | -1.2217871 | -5.7905186 | -0.3143353 |
| H | -4.5421525 | -6.4406635 | -1.0226945 |
| H | -3.8663714 | -5.2464906 | 0.0806532 |
| C | -3.4947526 | -4.8208924 | -2.0276381 |
| O | -1.3459618 | -4.6871960 | 0.2502179 |
| H | -2.4678076 | -4.4285856 | -2.0620202 |
| H | -3.6322899 | -5.3638903 | -2.9738872 |
| C | -4.4768974 | -3.6247275 | -1.9699812 |
| H | -5.0484475 | -3.5434805 | -2.9029711 |
| H | -5.2093622 | -3.7728432 | -1.1623582 |
| N | -3.8647761 | -2.3030211 | -1.7672457 |
| H | -3.7574861 | -1.6927276 | -2.5721747 |
| C | -3.5208500 | -1.7762049 | -0.5741098 |
| N | -3.3145130 | -2.5830196 | 0.4857028 |
| N | -3.4127401 | -0.4566010 | -0.4162300 |
| H | -2.7932889 | -3.4629553 | 0.3355524 |
| H | -3.0743311 | -2.0794269 | 1.3446288 |
| H | -3.6456187 | 0.1798966 | -1.1685750 |
| H | -2.9244734 | -0.1165215 | 0.4484159 |
| N | -0.0115298 | -6.2479419 | -0.7098097 |
| H | 0.0127870 | -7.1164068 | -1.2353690 |
| C | 1.1138517 | -5.3335549 | -0.8468321 |
| H | 1.4137243 | -4.9634011 | 0.1433091 |
| C | 2.3209517 | -6.0340386 | -1.5226301 |
| C | 0.6722488 | -4.1514772 | -1.7313158 |
| H | 2.6646440 | -6.8552865 | -0.8763887 |
| H | 1.9804968 | -6.4650842 | -2.4766950 |
| C | 3.4156930 | -5.0412164 | -1.7702432 |
| O | -0.0008776 | -4.3315691 | -2.7543681 |
| C | 3.5393704 | -4.2546384 | -2.8796216 |
| C | 4.3785768 | -4.5540891 | -0.8007520 |
| H | 2.9650938 | -4.2637931 | -3.8012439 |
| C | 5.0481957 | -3.4438327 | -1.3777148 |
| C | 4.7369522 | -4.9792608 | 0.4864096 |
| C | 6.0617334 | -2.7507150 | -0.7134972 |
| C | 5.7293019 | -4.2768879 | 1.1715168 |
| H | 4.2595998 | -5.8477463 | 0.9411181 |
| H | 6.5834583 | -1.9274768 | -1.1947109 |
| C | 6.3871532 | -3.1833051 | 0.5764534 |
| H | 6.0148051 | -4.5937439 | 2.1747217 |
| H | 7.1801822 | -2.6734763 | 1.1246990 |
| N | 1.1344761 | -2.9510608 | -1.3434636 |
| H | 1.6699977 | -2.8369846 | -0.4806332 |
| C | 0.8621262 | -1.7585364 | -2.1093767 |
| H | 0.9872496 | -1.9895888 | -3.1810296 |
| C | -0.6152448 | -1.3382612 | -1.8613056 |
| C | 1.8887249 | -0.7026077 | -1.6829509 |
| H | -1.1942570 | -2.2572049 | -2.0209184 |
| H | -0.6937234 | -1.0641087 | -0.8008401 |
| C | -1.1611875 | -0.2515892 | -2.7268413 |
| O | 2.6895549 | -0.9171284 | -0.7653892 |
| C | -1.6719045 | -0.3789536 | -3.9933132 |
| C | -1.2817363 | 1.1510517 | -2.3714812 |
| H | -1.7470817 | -1.2590650 | -4.6262969 |
| C | -1.8831197 | 1.8280358 | -3.4642163 |
| C | -0.8916153 | 1.8727104 | -1.2338465 |
| C | -2.1308975 | 3.2020923 | -3.4504277 |
| C | -1.1288194 | 3.2467116 | -1.2187348 |
| H | -0.3841132 | 1.3682930 | -0.4082787 |
| H | -2.5905597 | 3.6986103 | -4.3009867 |
| C | -1.7474076 | 3.8969271 | -2.3031749 |
| H | -0.8190973 | 3.8413288 | -0.3596628 |
| H | -1.9243864 | 4.9719777 | -2.2531481 |
| N | 1.8486359 | 0.4642880 | -2.3593149 |
| H | 1.1678926 | 0.6251205 | -3.1010562 |
| C | 2.5862570 | 1.6277478 | -1.8932677 |
| H | 2.4394751 | 1.7154736 | -0.8075388 |
| C | 4.1068866 | 1.5531564 | -2.2344471 |
| C | 1.9723973 | 2.8453713 | -2.5655516 |
| H | 4.3282011 | 0.5224224 | -2.5372916 |
| H | 4.3101304 | 2.1774699 | -3.1177763 |
| C | 5.0655089 | 1.9499823 | -1.1076829 |
| O | 1.3017138 | 2.8160960 | -3.5784123 |
| H | 6.0709890 | 2.0421989 | -1.5432504 |
| H | 4.8212079 | 2.9493963 | -0.7112679 |
| C | 5.1669268 | 0.9354485 | 0.0397874 |
| H | 5.0890722 | -0.0776659 | -0.3818316 |
| H | 6.1471798 | 1.0291927 | 0.5356823 |
| N | 4.1083635 | 1.1031792 | 1.0335764 |
| H | 3.7066376 | 2.0308412 | 1.1560833 |
| C | 3.5792963 | 0.1298198 | 1.7861007 |
| N | 4.0787233 | -1.1168408 | 1.8124785 |
| N | 2.5586723 | 0.4395699 | 2.6082743 |
| H | 4.7897123 | -1.3717597 | 1.1307080 |
| H | 3.3684240 | -1.8521819 | 1.9688897 |
| H | 1.8974934 | 1.1585609 | 2.3002782 |
| H | 2.1210290 | -0.3293060 | 3.1049935 |
| C | -3.4763119 | -8.7578686 | 0.2511207 |
| H | -3.4518994 | -9.4362046 | 1.1137030 |
| H | -4.5267535 | -8.4490798 | 0.0868267 |
| H | -3.1501914 | -9.3295600 | -0.6304111 |
| O | 2.3329819 | 3.9729201 | -1.9165204 |
| C | 1.8662125 | 5.2083439 | -2.5175099 |
| H | 2.2223945 | 6.0028467 | -1.8547066 |
| H | 2.2855135 | 5.3250867 | -3.5250119 |
| H | 0.7705565 | 5.2020500 | -2.5786431 |
| O | 2.5168599 | -5.1524021 | 2.5753432 |
| H | 2.8703134 | -4.4803726 | 1.9592988 |
| C | 1.1631451 | -4.7897779 | 2.8346118 |
| H | 0.4914723 | -5.1434332 | 2.0288539 |
| H | 0.8679155 | -5.2883719 | 3.7695863 |
| C | 1.0086571 | -3.2763369 | 2.9791717 |
| H | 1.5051268 | -2.9456747 | 3.9105882 |
| O | 1.6794141 | -2.6520548 | 1.8601856 |
| H | 1.1041159 | -1.8782042 | 1.5222217 |
| C | -0.4644315 | -2.9035131 | 3.0467546 |
| H | -0.9685674 | -3.5226860 | 3.8029578 |
| H | -0.9268830 | -3.0979509 | 2.0658927 |
| O | -0.6447664 | -1.5317722 | 3.4633260 |
| P | -0.8314316 | -0.3852340 | 2.3286370 |
| O | -2.2938688 | -0.1522451 | 2.0027793 |
| O | 0.1067125 | -0.6863504 | 1.1758534 |
| O | -0.2529789 | 0.8639798 | 3.1916968 |
| C | -1.0980137 | 1.9859635 | 3.4947876 |
| H | -2.0773518 | 1.6338490 | 3.8458412 |
| H | -0.6130911 | 2.5385365 | 4.3079904 |
| C | -1.2961609 | 2.8963856 | 2.2799289 |
| H | -1.2077543 | 2.2928354 | 1.3679945 |
| C | -2.6557611 | 3.5657032 | 2.2839639 |
| H | -2.7185367 | 4.3014574 | 1.4660733 |
| H | -3.4111314 | 2.7804958 | 2.1196513 |
| O | -0.3003561 | 3.9737355 | 2.2256192 |
| C | 0.9025336 | 3.7388038 | 1.6703097 |
| C | 1.7193239 | 5.0065952 | 1.5900360 |
| H | 1.2354022 | 5.6381812 | 0.8241558 |
| H | 1.6088095 | 5.5586480 | 2.5350707 |
| C | 3.1818831 | 4.7620241 | 1.2272273 |
| H | 3.6629042 | 4.1711004 | 2.0262913 |
| H | 3.2114673 | 4.1589647 | 0.3076197 |
| C | 3.9710080 | 6.0539006 | 1.0064057 |
| H | 3.9934474 | 6.6437622 | 1.9383126 |
| H | 3.4439748 | 6.6822353 | 0.2659955 |
| C | 5.4018867 | 5.8008512 | 0.5210642 |
| H | 5.9178211 | 5.1427018 | 1.2412871 |
| H | 5.3628358 | 5.2423669 | -0.4303375 |
| C | 6.2059926 | 7.0878900 | 0.3279981 |
| H | 6.2928978 | 7.6450168 | 1.2723492 |
| H | 7.2228332 | 6.8786433 | -0.0318214 |
| H | 5.7219337 | 7.7510643 | -0.4047474 |
| O | 1.2694423 | 2.6470627 | 1.2502909 |
| O | -2.8209097 | 4.1993111 | 3.5620832 |
| C | -4.0275504 | 4.7553599 | 3.9453105 |
| O | -4.0815558 | 5.2742755 | 5.0331663 |
| C | -5.1851653 | 4.6597162 | 2.9704474 |
| H | -4.8751587 | 5.0883626 | 2.0017984 |
| H | -5.3844997 | 3.5925385 | 2.7674957 |
| C | -6.4490383 | 5.3480440 | 3.4852687 |
| H | -6.2245556 | 6.4067588 | 3.6869634 |
| H | -6.7225620 | 4.9128349 | 4.4584688 |
| C | -7.6208831 | 5.2336277 | 2.5072470 |
| H | -7.3361584 | 5.6687967 | 1.5323435 |
| H | -7.8371429 | 4.1675892 | 2.3116185 |
| C | -8.8935073 | 5.9211242 | 3.0133872 |
| H | -8.6763458 | 6.9854038 | 3.2047258 |
| H | -9.1726595 | 5.4889546 | 3.9888744 |
| C | -10.0673468 | 5.8000455 | 2.0390047 |
| H | -10.9655470 | 6.3010423 | 2.4258890 |
| H | -9.8246660 | 6.2538876 | 1.0659151 |
| H | -10.3251892 | 4.7453728 | 1.8579850 |
| C | -2.6754071 | 1.0759416 | -5.7286237 |
| H | -2.7028782 | 0.1340666 | -6.3204372 |
| C | 4.7927582 | -2.2404811 | -3.5564557 |
| H | 4.2232786 | -2.3532764 | -4.5045288 |
| O | 5.5561467 | -1.3188871 | -3.3318537 |
| O | -3.0810813 | 2.1442447 | -6.1327775 |
| N | 4.5167439 | -3.2679603 | -2.6660436 |
| N | -2.1167508 | 0.8687307 | -4.4648550 |

**RForWForWR@GAG**

| N | -3.9093841 | -6.7129812 | 0.6649413 |
| --- | --- | --- | --- |
| H | -3.7051575 | -6.4744477 | 1.6386078 |
| C | -3.6566624 | -5.5371432 | -0.1625074 |
| H | -3.6881499 | -5.8593773 | -1.2192798 |
| C | -4.6496622 | -4.3563743 | 0.0163731 |
| C | -2.2520884 | -5.0356645 | 0.1835352 |
| H | -5.6096533 | -4.6402395 | -0.4439881 |
| H | -4.8375837 | -4.2308657 | 1.0955260 |
| C | -4.1602360 | -3.0216017 | -0.5614475 |
| O | -1.9929502 | -4.6345486 | 1.3217862 |
| H | -3.3648519 | -2.6190865 | 0.0863095 |
| H | -3.6999536 | -3.1523945 | -1.5533890 |
| C | -5.3048735 | -2.0010057 | -0.6482008 |
| H | -5.9491228 | -2.2223993 | -1.5114417 |
| H | -5.9473112 | -2.0690427 | 0.2424447 |
| N | -4.8736804 | -0.6100872 | -0.7661726 |
| H | -4.5376367 | -0.2811934 | -1.6688593 |
| C | -4.6191911 | 0.1876075 | 0.2991086 |
| N | -4.6799368 | -0.3152801 | 1.5415960 |
| N | -4.3772328 | 1.4853070 | 0.1008453 |
| H | -4.3615971 | -1.2800385 | 1.6985182 |
| H | -4.3072847 | 0.2737535 | 2.3058850 |
| H | -4.4237918 | 1.8672658 | -0.8377552 |
| H | -3.9076983 | 2.0844985 | 0.8160316 |
| N | -1.3495018 | -5.0443762 | -0.8351147 |
| H | -1.7416830 | -5.2146273 | -1.7583103 |
| C | -0.1401923 | -4.2255215 | -0.8515793 |
| H | 0.3088192 | -4.1992169 | 0.1496322 |
| C | 0.8456978 | -4.8340128 | -1.9010234 |
| C | -0.5200987 | -2.8215311 | -1.3307723 |
| H | 1.3158600 | -5.7215960 | -1.4582570 |
| H | 0.2351719 | -5.1828002 | -2.7489070 |
| C | 1.8849992 | -3.8900784 | -2.4347174 |
| O | -1.3161366 | -2.7014086 | -2.2840898 |
| C | 1.6580669 | -2.8650546 | -3.3100882 |
| C | 3.3022907 | -3.8664813 | -2.1254638 |
| H | 0.7275389 | -2.5443508 | -3.7684996 |
| N | 2.8464726 | -2.1518980 | -3.5497521 |
| C | 3.8867298 | -2.7890183 | -2.8471570 |
| C | 4.1175043 | -4.6592560 | -1.3072532 |
| C | 5.2544530 | -2.5066254 | -2.8046265 |
| C | 5.4826983 | -4.3806633 | -1.2503206 |
| H | 3.6899208 | -5.4688823 | -0.7157203 |
| H | 5.6723127 | -1.6802224 | -3.3738138 |
| C | 6.0442809 | -3.3279597 | -1.9943212 |
| H | 6.1266630 | -4.9844717 | -0.6108752 |
| H | 7.1156475 | -3.1377033 | -1.9311825 |
| N | 0.1143577 | -1.7735115 | -0.7701811 |
| H | 0.7571425 | -1.9017701 | 0.0390440 |
| C | -0.0736377 | -0.4995168 | -1.4264923 |
| H | -0.1294400 | -0.7200042 | -2.5031130 |
| C | -1.4331791 | 0.1890920 | -1.0548709 |
| C | 1.0821400 | 0.4659901 | -1.2016754 |
| H | -2.0859319 | -0.6142023 | -0.6851647 |
| H | -1.2875268 | 0.8938257 | -0.2246682 |
| C | -2.0697539 | 0.8570711 | -2.2341601 |
| O | 1.8333247 | 0.4327699 | -0.2275379 |
| C | -2.3988731 | 0.2430123 | -3.4165583 |
| C | -2.4013375 | 2.2617332 | -2.3838215 |
| H | -2.2920488 | -0.8027617 | -3.6944152 |
| N | -2.9106185 | 1.1895982 | -4.3225292 |
| C | -2.9182772 | 2.4489488 | -3.6929200 |
| C | -2.3345440 | 3.3520979 | -1.5063693 |
| C | -3.3400544 | 3.6951277 | -4.1582434 |
| C | -2.7642277 | 4.5991766 | -1.9542483 |
| H | -2.0178002 | 3.2240584 | -0.4722135 |
| H | -3.7284558 | 3.8132840 | -5.1664608 |
| C | -3.2501371 | 4.7659416 | -3.2651431 |
| H | -2.7442360 | 5.4349295 | -1.2554890 |
| H | -3.5842879 | 5.7523449 | -3.5891258 |
| N | 1.1659928 | 1.4106357 | -2.1822079 |
| H | 0.4696068 | 1.4061283 | -2.9322466 |
| C | 1.7766172 | 2.7176123 | -1.9790542 |
| H | 1.6185178 | 3.0220257 | -0.9319488 |
| C | 3.2943572 | 2.8178681 | -2.2791762 |
| C | 0.9909539 | 3.6909149 | -2.8618331 |
| H | 3.5103746 | 2.2730740 | -3.2115732 |
| H | 3.5109290 | 3.8790828 | -2.4651168 |
| C | 4.1866744 | 2.3278890 | -1.1217140 |
| O | 0.3127076 | 3.3564909 | -3.8154931 |
| H | 5.0741794 | 2.9765864 | -1.0478909 |
| H | 3.6352004 | 2.4309017 | -0.1736987 |
| C | 4.6506565 | 0.8715408 | -1.2415499 |
| H | 3.8027793 | 0.2322464 | -1.5058709 |
| H | 5.4026387 | 0.7608577 | -2.0366515 |
| N | 5.1836314 | 0.4016394 | 0.0434718 |
| H | 5.7310726 | 1.0732687 | 0.5741852 |
| C | 4.6238192 | -0.5996831 | 0.7710396 |
| N | 4.0721586 | -1.6404609 | 0.1674372 |
| N | 4.6985469 | -0.5655755 | 2.1147592 |
| H | 4.1974804 | -1.7450452 | -0.8339996 |
| H | 3.3273158 | -2.2238781 | 0.6314972 |
| H | 4.7023603 | 0.3401217 | 2.5758218 |
| H | 4.2289890 | -1.3473467 | 2.6251007 |
| C | -5.2406381 | -7.2969640 | 0.5296750 |
| H | -5.2970789 | -8.2009904 | 1.1505660 |
| H | -6.0798295 | -6.6324489 | 0.8165585 |
| H | -5.3982554 | -7.6035226 | -0.5161844 |
| O | 1.1632191 | 4.9537822 | -2.4403100 |
| C | 0.4515750 | 5.9681421 | -3.2017195 |
| H | 0.6429923 | 6.9070736 | -2.6748852 |
| H | 0.8363369 | 6.0049364 | -4.2288900 |
| H | -0.6201158 | 5.7343549 | -3.2164285 |
| C | 0.0131172 | 3.7797888 | 1.5962330 |
| C | 0.6720587 | 2.4459333 | 1.9424963 |
| C | 1.5664892 | 2.5914789 | 3.1809520 |
| C | 2.5308233 | 3.7821376 | 2.9270584 |
| C | 1.0907945 | 4.8486030 | 1.3984635 |
| H | 1.3215431 | 2.1414652 | 1.1021704 |
| H | 0.9852567 | 2.8211035 | 4.0856099 |
| H | 3.2019662 | 3.5138609 | 2.0657993 |
| C | 0.7057231 | -1.6955201 | 3.0611910 |
| C | -0.3868593 | 0.3737530 | 2.7844042 |
| C | -1.8386315 | -1.6670507 | 3.2553652 |
| C | -0.5809517 | -2.5044753 | 2.9408891 |
| H | -0.1382277 | 0.6063124 | 3.8403973 |
| H | -1.9556151 | -1.5675828 | 4.3492989 |
| H | -0.6497225 | -2.8241582 | 1.8913491 |
| H | 1.7592192 | 4.4689864 | 0.5874251 |
| H | 0.9087433 | -1.3936075 | 4.1029867 |
| O | -0.4176489 | 1.5106259 | 1.9887142 |
| O | 1.8490989 | 4.9872261 | 2.5975861 |
| C | -1.7809699 | -0.2486086 | 2.6739847 |
| H | -1.9823948 | -0.3329947 | 1.5897144 |
| O | 0.5717403 | -0.5276417 | 2.2344823 |
| O | -0.5690284 | -3.7235426 | 3.6817105 |
| O | -3.0075963 | -2.3434067 | 2.7342335 |
| H | -2.7318386 | -3.2493610 | 2.4630317 |
| O | -2.7509629 | 0.5744594 | 3.3203774 |
| H | -2.6581600 | 1.5006981 | 2.9568593 |
| H | -0.5441291 | 3.6635779 | 0.6537942 |
| O | -0.8760447 | 4.1208617 | 2.6733454 |
| O | 3.2734918 | 3.9776608 | 4.0796325 |
| C | 0.6056010 | 6.2119985 | 0.8868062 |
| H | 1.5011969 | 6.8408058 | 0.7570465 |
| H | 0.1823946 | 6.0200840 | -0.1211384 |
| O | -0.2843062 | 6.9321937 | 1.7044023 |
| H | -1.1621676 | 6.4936608 | 1.6392177 |
| C | 4.3174114 | 4.9440080 | 3.9327557 |
| H | 4.8836558 | 4.9290224 | 4.8709056 |
| H | 4.9882834 | 4.6754713 | 3.0939373 |
| H | 3.9096114 | 5.9505391 | 3.7585977 |
| C | -0.1817882 | -3.6188482 | 5.0543844 |
| H | -0.7296275 | -2.8248130 | 5.5922213 |
| H | -0.4311928 | -4.5839176 | 5.5122428 |
| H | 0.9020453 | -3.4421761 | 5.1612651 |
| C | 1.9587974 | -2.3870913 | 2.4878285 |
| O | 1.8794889 | -2.7688899 | 1.2701722 |
| O | 2.9885564 | -2.4078934 | 3.2071586 |
| S | -2.4808049 | 4.2835578 | 2.3066230 |
| O | -3.0408729 | 4.7568020 | 3.5485205 |
| O | -2.5076047 | 5.1941399 | 1.1464993 |
| O | -2.9261769 | 2.9053352 | 1.9124391 |
| C | 2.5120358 | 0.8244544 | 4.6481259 |
| O | 2.1909900 | 1.3491436 | 5.7024901 |
| N | 2.3484404 | 1.3759388 | 3.3970969 |
| H | 2.9774265 | -0.1872020 | 4.5844450 |
| H | 2.3364193 | 0.6996965 | 2.6309835 |
| C | 2.8936255 | -0.9268606 | -4.2017722 |
| H | 1.9021992 | -0.6491073 | -4.6208381 |
| C | -3.3219744 | 0.8789775 | -5.6153833 |
| H | -3.1889304 | -0.2052736 | -5.8309257 |
| O | 3.8865967 | -0.2274385 | -4.2946123 |
| O | -3.7708009 | 1.6777009 | -6.4126173 |

**R3BT3BTR@POPG**

N -2.7912345 -7.5052411 0.4628859

H -2.9395433 -7.0378150 1.3600236

C -2.7025291 -6.5019394 -0.5996347

H -2.5774445 -7.0510611 -1.5495055

C -3.8984333 -5.5415520 -0.7371046

C -1.4297221 -5.6760055 -0.3510009

H -4.8161902 -6.1309057 -0.8779695

H -4.0145613 -5.0155003 0.2239543

C -3.7426959 -4.5300828 -1.8873529

O -1.4620990 -4.5989969 0.2737142

H -2.7028224 -4.1792108 -1.9586268

H -3.9461307 -5.0336143 -2.8437428

C -4.6685942 -3.2969131 -1.7440015

H -5.2928382 -3.1701501 -2.6370514

H -5.3547770 -3.4323530 -0.8945325

N -3.9893594 -2.0063977 -1.5528226

H -3.8939362 -1.3859282 -2.3524262

C -3.5495216 -1.5284048 -0.3723898

N -3.3282406 -2.3678028 0.6586282

N -3.3607570 -0.2189234 -0.1941721

H -2.8744491 -3.2737082 0.4630374

H -3.0144904 -1.8972302 1.5134637

H -3.5951420 0.4438993 -0.9229962

H -2.8262010 0.0718522 0.6579820

N -0.2739159 -6.1687661 -0.8561619

H -0.3318787 -7.0125548 -1.4179214

C 0.8771169 -5.2972415 -1.0519960

H 1.3108264 -5.0237655 -0.0800368

C 1.9633851 -5.9825483 -1.9255674

C 0.4092050 -4.0342803 -1.7936083

H 2.4235692 -6.8019525 -1.3537861

H 1.4731179 -6.4148179 -2.8106918

C 2.9834184 -4.9682088 -2.3708048

O -0.3691846 -4.1056833 -2.7536837

C 2.9054396 -4.3043534 -3.5664763

C 4.0127583 -4.4170879 -1.5244715

H 2.1865522 -4.4860684 -4.3616948

C 4.6692217 -3.3082098 -2.1285363

C 4.4280568 -4.8321089 -0.2435453

C 5.7129050 -2.6311154 -1.4848890

C 5.4556056 -4.1532990 0.4025296

H 3.9589314 -5.6954976 0.2302026

H 6.2270992 -1.8040725 -1.9759277

C 6.1002220 -3.0617574 -0.2173213

H 5.7801907 -4.4810719 1.3901746

H 6.9300430 -2.5678277 0.2908845

N 0.9811015 -2.8923200 -1.3797665

H 1.5843670 -2.8524908 -0.5553032

C 0.7445566 -1.6536686 -2.0782054

H 0.7961278 -1.8430155 -3.1628881

C -0.6810425 -1.1426489 -1.7210805

C 1.8455393 -0.6834392 -1.6558072

H -1.3200922 -2.0295030 -1.8150656

H -0.6674636 -0.8441415 -0.6649165

C -1.2292354 -0.0556186 -2.5908455

O 2.5825430 -0.9211213 -0.6922611

C -1.8713501 -0.2870972 -3.7833280

C -1.1721137 1.3546996 -2.2886862

H -2.0199168 -1.2568300 -4.2554944

C -1.7913941 2.1479311 -3.2950819

C -0.5750273 1.9990257 -1.1855697

C -1.8293812 3.5460962 -3.2135837

C -0.6014414 3.3867828 -1.1178947

H -0.0762826 1.4033511 -0.4169981

H -2.3045417 4.1402158 -3.9942543

C -1.2296352 4.1558069 -2.1177738

H -0.1198736 3.8984995 -0.2864368

H -1.2428079 5.2431925 -2.0353343

N 1.9356027 0.4591293 -2.3712996

H 1.3009042 0.6593058 -3.1431086

C 2.7523051 1.5580845 -1.8886196

H 2.5560999 1.6932269 -0.8141147

C 4.2701740 1.2960573 -2.1111724

C 2.3067241 2.8121487 -2.6272808

H 4.4052939 0.2052940 -2.0923694

H 4.5401283 1.6314317 -3.1231799

C 5.2018910 1.9384691 -1.0736588

O 1.6426364 2.8200562 -3.6441842

H 6.2025991 2.0500955 -1.5173140

H 4.8707930 2.9599322 -0.8292859

C 5.3866107 1.1205774 0.2100559

H 5.6200920 0.0827954 -0.0658259

H 6.2535285 1.5063623 0.7728098

N 4.2148029 1.1259822 1.0799835

H 3.7445681 2.0183227 1.2394458

C 3.7200227 0.0840789 1.7651322

N 4.2267975 -1.1603471 1.6725634

N 2.7298727 0.3047054 2.6472386

H 4.7968434 -1.3766199 0.8571601

H 3.5187314 -1.8918487 1.8464698

H 2.1146176 1.1062310 2.4914902

H 2.2460412 -0.5163438 2.9968771

C -3.7996516 -8.5449474 0.2507213

H -3.7585856 -9.2563396 1.0857367

H -4.8404671 -8.1777331 0.1632506

H -3.5579925 -9.0983717 -0.6687815

O 2.7935089 3.9179110 -2.0222591

C 2.4688429 5.1747302 -2.6713327

H 2.9263611 5.9472113 -2.0456265

H 2.8839813 5.1984763 -3.6866029

H 1.3793957 5.2972380 -2.7207551

O 2.5455858 -5.1589462 2.2343928

H 2.8516618 -4.4371490 1.6493712

C 1.2210504 -4.8053201 2.6289276

H 0.4837710 -5.0900688 1.8544123

H 0.9919805 -5.3683574 3.5452584

C 1.1060228 -3.3020614 2.8913188

H 1.6451153 -3.0490191 3.8230624

O 1.7529409 -2.6169335 1.7951959

H 1.1731287 -1.8336261 1.4955283

C -0.3545761 -2.9043368 3.0397886

H -0.8425546 -3.5587662 3.7767197

H -0.8599445 -3.0299830 2.0693662

O -0.4933340 -1.5563937 3.5464122

P -0.7061979 -0.3458597 2.4881422

O -2.1727411 -0.0665165 2.2207558

O 0.1786324 -0.5918095 1.2804738

O -0.0752505 0.8400237 3.4069175

C -0.8161935 2.0576274 3.5995375

H -1.8248534 1.8261321 3.9665183

H -0.2861537 2.6254555 4.3733871

C -0.9106612 2.8794310 2.3054289

H -0.6341100 2.2318626 1.4638440

C -2.3003091 3.4183602 2.0485920

H -2.3003588 4.0580801 1.1519764

H -2.9474812 2.5443642 1.8710902

O 0.0087523 4.0259721 2.3077651

C 1.2616343 3.8283262 1.8663787

C 2.0433955 5.1168127 1.7899670

H 1.4425120 5.8278311 1.1991942

H 2.0890303 5.5520577 2.8011003

C 3.4302192 4.9275099 1.1773473

H 4.0559414 4.3303066 1.8617885

H 3.3230662 4.3401139 0.2527351

C 4.1392992 6.2433635 0.8524993

H 4.3168309 6.8188814 1.7768782

H 3.4757661 6.8716642 0.2314581

C 5.4644271 6.0273537 0.1144511

H 6.1243036 5.3963860 0.7340459

H 5.2702923 5.4501102 -0.8069870

C 6.1785540 7.3341998 -0.2355444

H 6.4118132 7.9132605 0.6701670

H 7.1229800 7.1490745 -0.7655145

H 5.5514102 7.9679106 -0.8805003

O 1.7009886 2.7328871 1.5267351

O -2.7139234 4.1592363 3.2065481

C -4.0281991 4.5549502 3.3689497

O -4.3010217 5.2046064 4.3480539

C -5.0242035 4.1259494 2.3078166

H -4.6558557 4.4444084 1.3173522

H -5.0406688 3.0221145 2.2747131

C -6.4296596 4.6692656 2.5594540

H -6.3905955 5.7689430 2.5899408

H -6.7612194 4.3559873 3.5607712

C -7.4332215 4.2050631 1.5011909

H -7.0875649 4.5169618 0.4988803

H -7.4644232 3.1004487 1.4815021

C -8.8481654 4.7433226 1.7344388

H -8.8165786 5.8454429 1.7539097

H -9.1917196 4.4320670 2.7348809

C -9.8467738 4.2733708 0.6744490

H -10.8539201 4.6702068 0.8636699

H -9.5419102 4.6023589 -0.3308248

H -9.9189024 3.1750550 0.6568184

S 4.0231826 -2.9862213 -3.7139216

S -2.4277242 1.1535167 -4.5795939

**R3BT3BTR@GAG**

N -3.8723697 -6.5809781 0.1194837

H -3.6491090 -6.4135315 1.1036468

C -3.6149507 -5.3529719 -0.6277855

H -3.6654242 -5.6007167 -1.7036897

C -4.5890375 -4.1795941 -0.3550870

C -2.1950056 -4.9036206 -0.2691709

H -5.5635264 -4.4270519 -0.8060315

H -4.7486780 -4.1126535 0.7338009

C -4.1041047 -2.8160888 -0.8660579

O -1.9039239 -4.6090459 0.8961432

H -3.3035038 -2.4516324 -0.2014917

H -3.6619648 -2.8936875 -1.8733414

C -5.2601242 -1.8037046 -0.8756296

H -5.9031019 -1.9707689 -1.7517952

H -5.8957446 -1.9529827 0.0097017

N -4.8591959 -0.4002574 -0.8847144

H -4.5607954 0.0139567 -1.7653079

C -4.6462762 0.3223298 0.2398562

N -4.6544099 -0.2874620 1.4372887

N -4.5082124 1.6477139 0.1528061

H -4.2594041 -1.2347117 1.5084232

H -4.3145398 0.2646880 2.2390851

H -4.5855820 2.0931972 -0.7551775

H -4.0258756 2.2066085 0.8910276

N -1.3255148 -4.8542266 -1.3064434

H -1.7225407 -4.9615643 -2.2372964

C -0.0142700 -4.2088842 -1.3013557

H 0.3291020 -4.0902645 -0.2663369

C 0.9512942 -5.0983179 -2.1218811

C -0.2024681 -2.8478996 -1.9941332

H 1.0260718 -6.0666548 -1.6047285

H 0.4697357 -5.2863897 -3.0942167

C 2.3238693 -4.5405979 -2.3672432

O -0.9087674 -2.7875839 -3.0182127

C 2.6861742 -3.9171696 -3.5298919

C 3.4330800 -4.6546185 -1.4492546

H 2.0451701 -3.7123327 -4.3841288

C 4.6390679 -4.1392623 -1.9944237

C 3.4436999 -5.1439027 -0.1297050

C 5.8320813 -4.1207740 -1.2630932

C 4.6215875 -5.1123464 0.6036301

H 2.5199161 -5.4996164 0.3267532

H 6.7482617 -3.7160372 -1.6941755

C 5.8098638 -4.6108627 0.0392027

H 4.6216307 -5.4567589 1.6372103

H 6.7237303 -4.5938266 0.6337504

N 0.3927182 -1.7699718 -1.4401851

H 0.9130566 -1.8600666 -0.5403203

C 0.0446393 -0.4829235 -2.0017587

H -0.0232341 -0.6164086 -3.0909247

C -1.3685598 -0.0231265 -1.4979589

C 1.0539727 0.6112638 -1.6799058

H -1.9124642 -0.9396135 -1.2346780

H -1.2505480 0.5571889 -0.5724490

C -2.1438279 0.7355877 -2.5318221

O 1.7801880 0.6034182 -0.6852719

C -2.5910781 0.1671812 -3.7006328

C -2.5027685 2.1271596 -2.4424008

H -2.4279750 -0.8652524 -4.0080977

C -3.2373178 2.5708128 -3.5763243

C -2.2614234 3.0311362 -1.3907016

C -3.7182059 3.8841930 -3.6665169

C -2.7496979 4.3286892 -1.4682683

H -1.7603532 2.6797950 -0.4879513

H -4.2831738 4.2145129 -4.5384907

C -3.4715705 4.7506631 -2.6050381

H -2.6164539 5.0008938 -0.6191884

H -3.8595506 5.7692771 -2.6447422

N 1.0287874 1.6482227 -2.5688425

H 0.3510577 1.6345653 -3.3345033

C 1.5077872 2.9799849 -2.2232701

H 1.3533227 3.1380695 -1.1447954

C 3.0004789 3.2650472 -2.5273162

C 0.6239034 3.9817656 -2.9676376

H 3.2169041 2.9655278 -3.5652101

H 3.1279193 4.3555350 -2.4777591

C 3.9793811 2.6082319 -1.5366356

O -0.0391314 3.7167065 -3.9518880

H 4.8515052 3.2681915 -1.4006963

H 3.4898348 2.5135536 -0.5556225

C 4.4792161 1.2173828 -1.9526669

H 3.6348264 0.5912068 -2.2583664

H 5.1682198 1.2907706 -2.8083040

N 5.1525509 0.5522595 -0.8292504

H 5.7718606 1.1450632 -0.2826911

C 4.6077015 -0.4866283 -0.1349752

N 3.9481201 -1.4493890 -0.7560928

N 4.8096089 -0.5654146 1.1924905

H 3.9734337 -1.5200591 -1.7678852

H 3.2787454 -2.0628143 -0.2193811

H 4.8915239 0.3039213 1.7121281

H 4.3622653 -1.3617069 1.7096708

C -5.2130992 -7.1371455 -0.0344560

H -5.2714546 -8.0834880 0.5196841

H -6.0388388 -6.4857015 0.3152730

H -5.3927070 -7.3640843 -1.0969933

O 0.7102494 5.1951434 -2.3937800

C -0.0632197 6.2456481 -3.0330580

H 0.0776271 7.1284433 -2.4033367

H 0.3104851 6.4216167 -4.0502221

H -1.1197652 5.9542933 -3.0728813

C -0.1595887 3.9471618 1.4909110

C 0.5876841 2.6145221 1.5813187

C 1.6356193 2.6769183 2.7022126

C 2.5244793 3.9241893 2.4360699

C 0.8448228 5.0798188 1.2610589

H 1.1358050 2.4466727 0.6367133

H 1.1714805 2.8098294 3.6905460

H 3.0653618 3.7712989 1.4621079

C 0.8566921 -1.5922878 2.5352363

C -0.3087656 0.4474472 2.3918292

C -1.6660255 -1.6679729 2.8766107

C -0.4026811 -2.4491463 2.4601066

H 0.0246561 0.6641806 3.4268449

H -1.7291114 -1.6333199 3.9788791

H -0.5201695 -2.7332416 1.4038890

H 1.3875608 4.8208361 0.3203555

H 1.1012629 -1.2999400 3.5708393

O -0.4356946 1.6037277 1.6295976

O 1.7729227 5.1272279 2.3422828

C -1.6907772 -0.2135002 2.3810603

H -1.9897408 -0.2394013 1.3145876

O 0.6213227 -0.4188170 1.7397203

O -0.3016737 -3.6917177 3.1537849

O -2.8426255 -2.3451175 2.3739351

H -2.5705111 -3.2441433 2.0738530

O -2.6185119 0.5287044 3.1656842

H -2.5987805 1.4795560 2.8573151

H -0.8438751 3.9151788 0.6323944

O -0.8992243 4.1179759 2.7122368

O 3.4286050 4.0314751 3.4801522

C 0.2538692 6.4699371 0.9899220

H 1.1035637 7.1417979 0.7860834

H -0.3284433 6.3723927 0.0494657

O -0.4990770 7.0633092 2.0180910

H -1.3711933 6.6078181 2.0407300

C 4.4142545 5.0476211 3.2810395

H 5.1167971 4.9620715 4.1179400

H 4.9565721 4.8901513 2.3283946

H 3.9613086 6.0499303 3.2749592

C 0.1640916 -3.6163429 4.5040177

H -0.3846370 -2.8677084 5.1024517

H -0.0141210 -4.6070193 4.9399810

H 1.2432586 -3.3921932 4.5528362

C 2.1019421 -2.2335934 1.8904734

O 1.9727633 -2.5885923 0.6714734

O 3.1660181 -2.2614943 2.5574505

S -2.5432249 4.2648401 2.5721036

O -2.9566030 4.4962061 3.9346542

O -2.7429970 5.3557281 1.5991857

O -2.9931952 2.9515071 2.0034339

C 2.8157549 0.8651073 3.9005451

O 2.6111309 1.3141711 5.0179731

N 2.4754200 1.4829760 2.7184685

H 3.2994313 -0.1222858 3.7209280

H 2.3929023 0.8632386 1.9106358

S 4.3709827 -3.4788395 -3.5942071

S -3.4484757 1.2748788 -4.7282743

**RnaINaIR@POPG**

N -2.8289762 -7.4720509 0.5440800

H -2.9708240 -7.0582140 1.4681980

C -2.7634963 -6.4080916 -0.4596200

H -2.6321542 -6.8985787 -1.4403649

C -3.9768564 -5.4631044 -0.5345477

C -1.5061967 -5.5743324 -0.1647227

H -4.8857407 -6.0581796 -0.7046747

H -4.0933870 -4.9966142 0.4571540

C -3.8429427 -4.3808546 -1.6209844

O -1.5560947 -4.5455781 0.5345923

H -2.8070159 -4.0161735 -1.6774395

H -4.0480581 -4.8242709 -2.6063584

C -4.7775617 -3.1677451 -1.3884044

H -5.4502181 -3.0192521 -2.2420104

H -5.4153531 -3.3412939 -0.5089295

N -4.1037159 -1.8782995 -1.1852069

H -4.0136829 -1.2479306 -1.9783865

C -3.6014881 -1.4449755 -0.0130615

N -3.3640024 -2.3120885 0.9903596

N -3.3718942 -0.1440560 0.1899799

H -2.9474236 -3.2275068 0.7602576

H -2.9853079 -1.8671632 1.8337902

H -3.5970823 0.5398286 -0.5223359

H -2.7753232 0.1026891 1.0114775

N -0.3405469 -6.0066495 -0.7015491

H -0.3884590 -6.7800206 -1.3582411

C 0.7844314 -5.0879664 -0.8154458

H 1.0809913 -4.7597872 0.1894727

C 1.9931697 -5.7673350 -1.5050284

C 0.3112943 -3.8628631 -1.6188830

H 2.1572223 -6.7323179 -1.0028775

H 1.7323003 -5.9836255 -2.5522175

O -0.5052963 -3.9657229 -2.5444918

N 0.8922675 -2.7063097 -1.2674791

H 1.5601001 -2.6516655 -0.4971714

C 0.6063769 -1.4811779 -1.9706747

H 0.5663752 -1.6935214 -3.0513010

C -0.7790060 -0.9506004 -1.4991762

C 1.7511834 -0.5176219 -1.6643779

H -1.4129438 -1.8467043 -1.4808384

H -0.6695827 -0.6153875 -0.4622556

O 2.6019366 -0.7841455 -0.8088215

N 1.7501148 0.6501051 -2.3433445

H 1.0266130 0.8684835 -3.0276979

C 2.5939558 1.7512247 -1.9108445

H 2.5055348 1.8468155 -0.8173260

C 4.0914034 1.5619150 -2.3068892

C 2.0468433 3.0177416 -2.5514071

H 4.1975242 0.5269012 -2.6575187

H 4.3206234 2.2069918 -3.1686215

C 5.1178249 1.8255935 -1.1980347

O 1.2944620 3.0485995 -3.5044340

H 6.1059090 1.9187292 -1.6724348

H 4.9345155 2.7992033 -0.7147474

C 5.2351538 0.7161683 -0.1416076

H 5.0558251 -0.2501192 -0.6385417

H 6.2563312 0.7061990 0.2739074

N 4.2790483 0.8600610 0.9530260

H 3.8760745 1.7788593 1.1348242

C 3.7985551 -0.1476564 1.6935520

N 4.2326432 -1.4146028 1.5549196

N 2.9161064 0.1285532 2.6721176

H 4.7550722 -1.6475956 0.7140515

H 3.5188657 -2.1239801 1.7948370

H 2.2914925 0.9270391 2.5360425

H 2.4642959 -0.6667291 3.1117377

C -3.8291151 -8.5088370 0.2847020

H -3.7609207 -9.2739141 1.0691256

H -4.8760426 -8.1509162 0.2439791

H -3.6010955 -8.9965220 -0.6747127

O 2.5649495 4.1088534 -1.9467292

C 2.1672113 5.3819847 -2.5185926

H 2.6364210 6.1405488 -1.8850506

H 2.5246107 5.4626697 -3.5530015

H 1.0736571 5.4726462 -2.5046541

O 2.6285489 -5.3478922 2.4544193

H 2.8823748 -4.7235333 1.7464709

C 1.3156151 -4.9711892 2.8590550

H 0.5563311 -5.2943158 2.1199460

H 1.1100314 -5.4910959 3.8058146

C 1.2094727 -3.4585890 3.0587026

H 1.8046650 -3.1690929 3.9448946

O 1.7810118 -2.8175518 1.8950681

H 1.2023457 -2.0091211 1.6539739

C -0.2398385 -3.0514729 3.2831601

H -0.6866112 -3.6897668 4.0591692

H -0.8028301 -3.1882902 2.3468550

O -0.3433074 -1.6954716 3.7790816

P -0.5567439 -0.4812366 2.7273169

O -2.0223128 -0.1449831 2.5301163

O 0.2626055 -0.7587281 1.4810407

O 0.1603955 0.6863288 3.6063206

C -0.5563261 1.9023496 3.8797255

H -1.5400964 1.6698199 4.3087042

H 0.0291365 2.4494543 4.6281751

C -0.7296025 2.7535568 2.6141380

H -0.5375869 2.1169835 1.7405588

C -2.1199690 3.3331606 2.4761389

H -2.1680760 4.0022448 1.6025392

H -2.7992460 2.4804367 2.3165281

O 0.2222925 3.8716918 2.5710108

C 1.4283062 3.6528600 2.0190582

C 2.2408216 4.9208539 1.9287325

H 1.6221737 5.6648394 1.4002119

H 2.3656729 5.3230304 2.9468778

C 3.5762961 4.7149789 1.2175898

H 4.2263197 4.0708696 1.8333012

H 3.3867726 4.1732083 0.2788149

C 4.3047031 6.0210706 0.8965111

H 4.5781502 6.5438538 1.8287626

H 3.6165761 6.7006858 0.3622924

C 5.5552534 5.7979913 0.0409111

H 6.2312247 5.0993572 0.5633686

H 5.2589628 5.2932376 -0.8955799

C 6.3021505 7.0931112 -0.2828553

H 6.6366629 7.5979607 0.6354229

H 7.1899686 6.9032739 -0.9015093

H 5.6569721 7.7965076 -0.8305476

O 1.8014158 2.5602265 1.6013352

O -2.4375734 4.0427244 3.6831718

C -3.7275291 4.4724125 3.9359732

O -3.9259544 5.0820710 4.9577729

C -4.7900304 4.1293672 2.9085918

H -4.4601677 4.4861941 1.9176977

H -4.8451717 3.0298483 2.8212511

C -6.1639497 4.7012396 3.2542393

H -6.0878202 5.7960118 3.3405272

H -6.4616082 4.3443417 4.2515652

C -7.2263130 4.3246814 2.2180516

H -6.9120305 4.6785137 1.2193480

H -7.2939989 3.2241403 2.1422577

C -8.6122126 4.8928697 2.5390460

H -8.5429699 5.9907431 2.6151299

H -8.9246969 4.5388648 3.5353794

C -9.6697968 4.5127449 1.5001554

H -10.6533865 4.9318292 1.7540161

H -9.3964278 4.8847701 0.5007521

H -9.7815669 3.4199617 1.4285102

C 3.2615256 -4.9427862 -1.3971315

C 3.5932000 -3.9045635 -2.3291192

C 4.1093017 -5.1796462 -0.3238866

C 2.8147512 -3.6164545 -3.4842686

C 4.7677799 -3.1045643 -2.0921376

C 5.2650279 -4.3990177 -0.0969131

H 3.8822432 -5.9988094 0.3608064

C 3.1539994 -2.5902053 -4.3412237

H 1.9200215 -4.2017945 -3.6953648

C 5.0928533 -2.0614000 -3.0004306

C 5.5838931 -3.3729580 -0.9619482

H 5.9040942 -4.6229174 0.7578508

C 4.3033597 -1.8016747 -4.0992236

H 2.5374085 -2.3925940 -5.2188377

H 5.9946325 -1.4730183 -2.8169792

H 6.4873700 -2.7771914 -0.8094206

H 4.5669383 -1.0005875 -4.7907797

C -1.4380719 0.0637965 -2.3922497

C -1.4226179 1.4690252 -2.1099405

C -2.1023374 -0.3892883 -3.5265152

C -0.7318145 2.0254290 -0.9949771

C -2.1085319 2.3676762 -3.0037632

C -2.7792638 0.4934752 -4.3978020

H -2.0864991 -1.4611140 -3.7436919

C -0.7095757 3.3898946 -0.7899496

H -0.1867958 1.3680168 -0.3148994

C -2.0824531 3.7643927 -2.7419372

C -2.7873687 1.8474884 -4.1365102

H -3.2877643 0.0998722 -5.2787335

C -1.3967981 4.2684598 -1.6595628

H -0.1469229 3.8045710 0.0439615

H -2.6082559 4.4339098 -3.4249919

H -3.3031964 2.5390038 -4.8049677

H -1.3790929 5.3428761 -1.4716369

**RNalNalR@GAG**

N -3.8208255 -6.5621520 0.1394915

H -3.5973822 -6.4219800 1.1278268

C -3.5767990 -5.3098385 -0.5707831

H -3.6274984 -5.5253128 -1.6535977

C -4.5612934 -4.1544548 -0.2603150

C -2.1608425 -4.8563818 -0.2013967

H -5.5368152 -4.4004405 -0.7098187

H -4.7120440 -4.1173583 0.8312344

C -4.0947952 -2.7735522 -0.7403736

O -1.8704679 -4.5980556 0.9731385

H -3.2887951 -2.4194542 -0.0766526

H -3.6657435 -2.8211880 -1.7551472

C -5.2593123 -1.7708405 -0.7107046

H -5.9147460 -1.9265582 -1.5795441

H -5.8796554 -1.9421810 0.1814776

N -4.8681633 -0.3648494 -0.7017657

H -4.5894869 0.0644974 -1.5818850

C -4.6204335 0.3334726 0.4308743

N -4.6198697 -0.2963982 1.6180531

N -4.4548077 1.6569404 0.3632152

H -4.2444551 -1.2521178 1.6639844

H -4.2487894 0.2327506 2.4234254

H -4.5359911 2.1197294 -0.5354052

H -3.9730092 2.2000626 1.1133212

N -1.2967314 -4.7589384 -1.2385155

H -1.6900116 -4.8512202 -2.1727133

C 0.0087868 -4.1007865 -1.2168913

H 0.3376118 -3.9790907 -0.1780956

C 0.9956807 -4.9747769 -2.0273964

C -0.1971844 -2.7426657 -1.9117555

H 1.0383097 -5.9588621 -1.5399656

H 0.5416500 -5.1292120 -3.0188423

O -0.8774891 -2.7046692 -2.9536361

N 0.3448658 -1.6455569 -1.3409445

H 0.8684173 -1.7265696 -0.4447992

C -0.0280713 -0.3622479 -1.8994361

H -0.0915696 -0.4933183 -2.9894819

C -1.4478577 0.0745690 -1.3905965

C 0.9698873 0.7400591 -1.5691358

H -1.9720058 -0.8568103 -1.1390725

H -1.3350794 0.6327758 -0.4530764

O 1.7046380 0.7204839 -0.5807740

N 0.9297262 1.7919366 -2.4392549

H 0.2455718 1.7892604 -3.1989996

C 1.4138768 3.1171887 -2.0760303

H 1.2736586 3.2557465 -0.9927554

C 2.9018916 3.4063202 -2.3996011

C 0.5285903 4.1358545 -2.7943299

H 3.0968143 3.1348519 -3.4495937

H 3.0334006 4.4948429 -2.3229885

C 3.8986535 2.7204732 -1.4481742

O -0.1327165 3.8960357 -3.7860672

H 4.7772447 3.3727903 -1.3158990

H 3.4307288 2.6034881 -0.4589821

C 4.3826012 1.3388344 -1.9104617

H 3.5295422 0.7176988 -2.2022351

H 5.0446468 1.4315238 -2.7853483

N 5.0908025 0.6500061 -0.8231058

H 5.7219092 1.2348960 -0.2811603

C 4.5518678 -0.3913397 -0.1250646

N 3.8829202 -1.3489393 -0.7425023

N 4.7666428 -0.4707992 1.1997606

H 3.9321992 -1.4517016 -1.7506675

H 3.2309374 -1.9739489 -0.1943336

H 4.8511430 0.3961154 1.7230952

H 4.3557990 -1.2827740 1.7219508

C -5.1559387 -7.1270417 -0.0294404

H -5.2034095 -8.0911811 0.4942915

H -5.9877852 -6.4958445 0.3424468

H -5.3354837 -7.3218467 -1.0983664

O 0.6195928 5.3359285 -2.1927560

C -0.1400801 6.4067033 -2.8131472

H -0.0274506 7.2635119 -2.1429563

H 0.2674756 6.6282623 -3.8082800

H -1.1924614 6.1118527 -2.9031987

C -0.1701981 3.9499934 1.7198137

C 0.5895463 2.6239065 1.7732735

C 1.6555947 2.6753058 2.8771246

C 2.5316234 3.9314419 2.6097884

C 0.8208410 5.0929645 1.4822571

H 1.1215201 2.4782799 0.8158120

H 1.2084797 2.7923897 3.8751825

H 3.0545174 3.7935453 1.6237587

C 0.9025348 -1.5966292 2.6371630

C -0.2818248 0.4368737 2.5542866

C -1.6164052 -1.6997343 3.0095108

C -0.3520027 -2.4606898 2.5600824

H 0.0616975 0.6340049 3.5901418

H -1.6649490 -1.6873416 4.1130025

H -0.4822018 -2.7212555 1.4994133

H 1.3457410 4.8497858 0.5270499

H 1.1627658 -1.3278936 3.6753069

O -0.4277052 1.6071849 1.8192214

O 1.7713137 5.1312323 2.5446600

C -1.6570516 -0.2363253 2.5421497

H -1.9630935 -0.2442200 1.4769342

O 0.6482249 -0.4059281 1.8732020

O -0.2305393 -3.7183621 3.2227090

O -2.7943211 -2.3745440 2.5085028

H -2.5196939 -3.2638935 2.1825581

O -2.5853210 0.4833049 3.3476986

H -2.5678689 1.4435751 3.0682162

H -0.8746707 3.9132851 0.8784823

O -0.8820505 4.0991394 2.9600026

O 3.4547980 4.0323259 3.6375726

C 0.2194784 6.4844466 1.2429302

H 1.0634267 7.1621330 1.0343907

H -0.3774231 6.3998614 0.3102288

O -0.5166717 7.0596274 2.2928867

H -1.3842359 6.5955497 2.3304597

C 4.4319272 5.0550287 3.4309022

H 5.1509353 4.9634730 4.2530681

H 4.9565594 4.9104737 2.4663591

H 3.9746587 6.0552856 3.4446543

C 0.2544453 -3.6705992 4.5672591

H -0.2900716 -2.9392930 5.1905239

H 0.0887080 -4.6720085 4.9832942

H 1.3328562 -3.4405252 4.6053997

C 2.1393488 -2.2077064 1.9497769

O 1.9816236 -2.5238552 0.7240291

O 3.2198330 -2.2424267 2.5894026

S -2.5299164 4.2393312 2.8605777

O -2.9129980 4.4359828 4.2372746

O -2.7580541 5.3527886 1.9190711

O -2.9858571 2.9379836 2.2710679

C 2.8711018 0.8550010 4.0277613

O 2.6923952 1.2906000 5.1547263

N 2.5019595 1.4859032 2.8608552

H 3.3534684 -0.1286541 3.8243181

H 2.3965846 0.8720268 2.0510390

C 2.3839638 -4.4148862 -2.2279212

C 3.4219663 -4.6195922 -1.2624650

C 2.6707038 -3.7276059 -3.3969134

C 3.1847848 -5.2119392 0.0067723

C 4.7557407 -4.1719867 -1.5659713

C 3.9783025 -3.2717493 -3.6872529

H 1.8696970 -3.5563374 -4.1192382

C 4.1978919 -5.3444192 0.9314596

H 2.1769197 -5.5218187 0.2780536

C 5.7812524 -4.3448439 -0.5994445

C 5.0072754 -3.5160083 -2.8006847

H 4.1719363 -2.7545551 -4.6284111

C 5.5087600 -4.9110776 0.6269197

H 3.9827740 -5.7643677 1.9138886

H 6.7889970 -3.9978036 -0.8378872

H 6.0246393 -3.1879442 -3.0247104

H 6.2994402 -5.0165787 1.3707075

C -2.2574034 0.8067432 -2.4327731

C -2.5874302 2.1958789 -2.3317426

C -2.7076689 0.0782575 -3.5291489

C -2.1952670 3.0083251 -1.2312968

C -3.3991812 2.7949030 -3.3617803

C -3.4821953 0.6757119 -4.5468431

H -2.4355254 -0.9787516 -3.5963177

C -2.6086754 4.3199318 -1.1244834

H -1.6157412 2.5654696 -0.4209412

C -3.7857135 4.1570918 -3.2342894

C -3.8230324 2.0099267 -4.4642120

H -3.8107795 0.0743886 -5.3956129

C -3.4135140 4.8992167 -2.1351424

H -2.3698135 4.8988670 -0.2308604

H -4.4047504 4.5982169 -4.0181182

H -4.4285997 2.4789923 -5.2421363

H -3.7478742 5.9319870 -2.0277059

**References**

[49] Becke, A. D. (1988). Density-functional exchange-energy approximation with correct asymptotic behavior. Physical Review A, 38(6), 3098–3100. <https://doi.org/10.1103/PhysRevA.38.3098>

[50] Perdew, J. P. (1986). Density-functional approximation for the correlation energy of the inhomogeneous electron gas. Physical Review B, 33 (12), 8822–8824. <https://doi.org/10.1103/PhysRevB.33.8822>

[51] Weigend, F., & Ahlrichs, R. (2005). Balanced basis sets of split valence, triple zeta valence and quadruple zeta valence quality for H to Rn: Design and assessment of accuracy. Physical Chemistry Chemical Physics, 7(18), 3297–3305. <https://doi.org/10.1039/B508541A>

[52] Grimme, S., Antony, J., Ehrlich, S., & Krieg, H. (2010). A consistent and accurate ab initio parametrization of density functional dispersion correction (DFT-D) for the 94 elements H-Pu. The Journal of Chemical Physics, 132 (15), 154104. <https://doi.org/10.1063/1.3382344>

[53] Furche, F., Ahlrichs, R., Hättig, C., Klopper, W., Sierka, M., & Weigend, F. (2014). Turbomole. Wiley Interdisciplinary Reviews: Computational Molecular Science, 4 (2), 91–100. <https://doi.org/10.1002/wcms.1162>

[54] Klamt, A., & Schüürmann, G. (1993). COSMO: A new approach to dielectric screening in solvents with explicit expressions for the screening energy and its gradient. Journal of the Chemical Society, Perkin Transactions 2, 5, 799–805. <https://doi.org/10.1039/P29930000799>

[55] Klamt, A. (1995). Conductor-like screening model for real solvents: A new approach to the quantitative calculation of solvation phenomena. The Journal of Physical Chemistry, 99 (7), 2224–2235. <https://doi.org/10.1021/j100007a062>
